# The effects of standardized cannabis products in healthy volunteers and patients: a systematic literature review

**DOI:** 10.3389/fphar.2024.1411631

**Published:** 2024-10-17

**Authors:** Nadia A. Leen, Mikael A. Kowal, Albert Batalla, Matthijs G. Bossong

**Affiliations:** ^1^ Bedrocan International BV, Clinical Research Unit, Utrecht, Netherlands; ^2^ Department of Psychiatry, UMC Utrecht Brain Center, University Medical Center Utrecht, Utrecht University, Utrecht, Netherlands

**Keywords:** tetrahydrocannabinol (THC), cannabidiol (CBD), Bedrocan, medicinal cannabis, chronic pain, side effects

## Abstract

**Introduction:**

There is growing recognition of the potential of cannabis to treat various medical conditions and symptoms, such as chronic pain, spasticity, and epilepsy. However, one of the biggest challenges is the assurance of a standardized cannabis product that contains a consistent amount of its main psychoactive substances delta-9-tetrahydrocannabinol (THC) and cannabidiol (CBD), and which is compliant with predetermined specifications for these compounds. This is crucial not only to ensure consistent cannabis quality and dosage for patients but also to effectively translate research findings into clinical practice.

**Methods:**

This systematic literature review provides an overview of the effects of standardized cannabis products from Bedrocan, a leading Dutch producer of pharmaceutical-quality standardized medicinal cannabis.

**Results:**

Cannabis administration to healthy volunteers induces dose-dependent acute effects, such as rapidly rising THC and CBD blood concentrations, the subjective experience of high and anxiety, slower reaction time and impaired attention, learning and working memory. Patient studies suggest that treatment with medicinal cannabis reduces pain intensity across a broad range of chronic pain-related medical conditions. Medicinal cannabis showed a mild safety profile, with minor and transient side effects, such as feeling high, coughing and mental confusion. The strength of acute effects, the experience of side effects and the drop-out rate in patient studies may depend on cannabis dose, cannabis composition (CBD:THC ratio), and cannabis use history of participants.

**Conclusion:**

Safety and efficacy of standardized medicinal cannabis products should be further investigated in randomized clinical trials with sufficient sample size, with particular focus on cannabis dose and composition, age and differences between males and females.

## 1 Introduction

Cannabis is the most used illicit drug with an estimation of 192 million users worldwide (United Nations Office on Drugs and Labor, 2021). Beyond its recreational use, there is a growing recognition of its potential to relieve various medical conditions and symptoms such as chronic pain, spasticity, and epilepsy (Bilbao and Spanagel, 2022). However, one of the biggest challenges is the assurance of a standardized cannabis product that contains a consistent amount of the main psychoactive substances delta-9-tetrahydrocannabinol (THC) and cannabidiol (CBD) (Cooper et al., 2021; Romero-Sandoval et al., 2018). The availability of standardized cannabis products is crucial not only to ensure consistent cannabis quality and dosage for patients treating their medical conditions over time, but also in translating research findings on its safety and efficacy from healthy volunteers, to patient populations and into clinical practice.

The cannabis plant is often categorized as a single drug, but the plant contains over 500 chemicals and >100 unique cannabinoids of which the two most studied are THC and CBD (Pertwee, 2014). The main reason for its recreational use is its broad range of acute psychotropic effects, such as feeling high, relaxation and euphoria (Green et al., 2004; Mané et al., 2015). However, it is also demonstrated that THC can cause unwanted side effects such as anxiety, paranoia and impairing effects on the cognitive domains of learning, memory and attention (Karila et al., 2014; Kroon et al., 2021; Zhornitsky et al., 2021). CBD on the other hand does not possess these intoxicating properties. It is thought that CBD may attenuate some of the negative effects associated with THC, such as anxiety and psychosis-like symptoms, although inconsistent findings have been reported (Freeman et al., 2019a; Niesink and van Laar, 2013). The acute effects of both THC and CBD are therefore important to consider when selecting cannabis products for the treatment of different medical conditions.

In the Netherlands, there are currently only two approved cannabis products for a specific medical indication: Sativex^®^, a CBD/THC sublingual spray prescribed for the treatment of spasticity symptoms in multiple sclerosis (Baratta et al., 2022), and Epidiolex^®^, a CBD-only oral solution indicated for the treatment of seizures associated with rare childhood epilepsy syndromes such as Lennox-Gastaut and Dravet syndrome (Arzimanoglou et al., 2020). In addition, for a variety of other medical conditions and symptoms including chronic pain, neuropathic pain and sleep problems, standardized medicinal cannabis products from Bedrocan, the world’s most experienced producer of standardized medicinal cannabis based in the Netherlands, are prescribed through a special access program by medical professionals (Ekhart et al., 2023). They have been cultivating standardized cannabis varieties on behalf of the Dutch government since 2003. Through a combination of stable plant genetics and state of the art production techniques, Bedrocan has the capacity to produce pharmaceutical-quality cannabis products according to guidelines for Good Medicinal Cannabis Cultivation Practice (GMCCP) and Good Manufacturing Practice (GMP) (Quality standards: GMP and GMCCP, 2024). Currently Bedrocan cultivates five different cannabis varieties with different THC and CBD composition and unique terpene and minor cannabinoid profiles. Bedrocan^®^ (THC 22%; CBD <1.0%), Bedrobinol^®^ (THC 13.5%; CBD <1.0%), Bediol^®^ (THC 6.3%; CBD 8%), Bedica^®^ (THC 14%; CBD <1.0%) and Bedrolite^®^ (THC <1.0%; CBD 7.5%) are from sativa plants, and Bedica^®^ (THC 14%; CBD <1.0%) from indica plants (Cannabis Products by Bedrocan, Bedrocan 2024). Although Bedrocan products have not yet received regulatory approval to be prescribed for a specific medical indication, these high-quality standardized cannabis products have become widely available for patients, pharmacies, and clinical research.

Over the years, an increasing number of human studies have been performed with administration of Bedrocan standardized cannabis products, both in healthy volunteers (e.g., Lawn et al., 2023; Oliver et al., 2023; van Dam et al., 2023) and in patients, primarily those with a pain-related medical condition (e.g., Aviram et al., 2022; Nunnari et al., 2022). Although some of these studies have been included in excellent reviews that describe the acute effects of cannabis in healthy volunteers and the impact of medicinal cannabis treatment in patients (e.g., Fisher et al., 2021; Kroon et al., 2021), the aim of the current systematic literature review is to provide a detailed overview of studies that investigated the effects of Bedrocan standardized cannabis products in both healthy volunteers and patients. This will provide more insight into the safety and efficacy of standardized cannabis, which unfortunately is still uncommon. Knowledge about cannabis with a stable chemical composition is crucial for medical professionals, especially when considering the number of active compounds present in cannabis (Pertwee, 2014). In this way patients are guaranteed to get consistent cannabis quality and dosage for treating their medical conditions over time but it is also important in translating research on its efficacy and safety into clinical practice. Better understanding of standardized medicinal cannabis products will assist medical professionals in the selection of products and dosages, particularly in the absence of standardized dosing regimens for various medical conditions treated with medicinal cannabis (Bhaskar et al., 2021; Carter et al., 2004). The reviewed papers addressed the acute effects of standardized cannabis in healthy volunteers, in particular on pharmacokinetics, subjective experiences, cognitive function, and the influence of CBD:THC ratios on these acute outcome measures. Studies in patients examined the impact of medicinal cannabis treatment on pain-related and other medical conditions, but also provide the opportunity to systematically review reported side effects associated with standardized medicinal cannabis treatment.

## 2 Methods

### 2.1 Search strategy and data inclusion

This review was conducted in line with the Preferred Reporting Items for Systematic Reviews and Meta-analyses (PRISMA) guidelines (Page et al., 2021). An electronic literature search was performed on 4 December 2023, using the following criteria in the PubMed database (Title/Abstract): (“cannabis” OR “marijuana” OR “marihuana” OR “THC’ OR “tetrahydrocannabinol” OR “CBD” OR “Cannabidiol”) AND (“placebo” OR “Bedrocan” OR “medical” OR “medicinal”). Reports in English and studies with human participants were additional filters, resulting in a total of 6.439 papers. Titles, abstracts, and methods (information about drug administration) were screened for eligibility by a first (NL) and second reviewer (MB or MK). The only requirement for inclusion in the current literature review was the use of one of the available Bedrocan cannabis varieties (Bedrocan^®^, Bedrobinol^®^, Bediol^®^, Bedica^®^ and/or Bedrolite^®^). Review articles, book chapters and commentaries were excluded. Both studies in healthy volunteers and patients were included and there was no exclusion based on publication year. Finally, references of included papers were examined to ensure the inclusion of additional relevant studies. All papers were read by NL, and an overview of the results according to the PICO framework (Schardt et al., 2007) was discussed between NL and MB. Based on this discussion, it was decided that research in healthy volunteers could be divided into four topics; every included paper presented data of at least one of the selected topics. Research topics that were selected were: 1) pharmacokinetics of THC and CBD, 2) acute subjective experiences, 3) impact on cognitive assessments, and 4) influence of CBD:THC ratios on acute outcome measures. Additionally, a division between studies in patients and case studies was made. Data was extracted by NL, including study design, sample size, mean age, sex, Bedrocan variety, THC and CBD content, route of administration, dosing regimen, and outcome measures per topic in the healthy volunteer studies. For the patient studies, data about indication, primary outcome measure, and side effects were also extracted. Since we aimed to include all types of studies in our systematic review to provide a comprehensive overview of studies conducted with Bedrocan products, we did not use a risk of bias tool. This is because such tools are typically designed for randomized controlled trials (RCTs), and there is currently no gold standard for assessing the risk of bias for non-randomized studies (Farrah et al., 2019; Quigley et al., 2019).

## 3 Results

A total of 60 papers were identified and included in the current systematic literature review. 49 of these papers were found through the electronic literature search in PubMed and 11 through references of the selected papers, see also Supplementary Material and Supplementary Figure S1 for the PRISMA flow diagram and an overview of the included articles.

### 3.1 The acute effects of standardized cannabis in healthy volunteers

37 papers (38 studies; Mokrysz et al. (2021) included two studies in their paper) reported on the acute effects of standardized cannabis in healthy volunteers. In general, 21 studies (55%) administered the cannabis variety Bedrobinol^®^, 16 studies (42%) Bedrocan^®^, 9 (24%) studies Bedrolite^®^, and 5 studies (13%) used Bediol^®^. Please note that because studies can use more than one variety the percentage does not add up to 100%. 31 studies (82%) included an additional placebo condition. The route of cannabis administration was for 27 studies (71%) through vapor inhalation and for 11 studies (29%) through smoking. Lastly, 30 studies (79%) administered a single dose of cannabis (12 studies (40%) in a single session and 18 (60%) in multiple sessions), 7 studies administered multiple dosages of cannabis (4 (57%) in a single session and 3 (43%) in multiple sessions), and in one study participants received either a single cannabis administration or 3 successive administrations in a single session. Research topics addressed in these papers could be subdivided into the following four categories: 1) pharmacokinetics of THC and CBD, 2) acute subjective experiences, 3) impact on cognitive assessments, and 4) influence of CBD:THC ratios on acute outcome measures. Papers related to each topic are summarized in Tables 1–4. Papers can be included in several tables due to the report of multiple outcome measures in one study. In case papers presented data derived from the same study, these papers were grouped together in the first column of Tables 1–4.

**TABLE 1:** Overview of studies investigating pharmacokinetics after cannabis administration.

First author	Study design	Groups (if applicable)	N	Mean age (SD)	M/F	Study medication^a^	Route	Frequency	Outcome measure(s)	Concentrations	Main effects in mean (SD) ng/mL	Time after administration (in minutes)
**Hutten et al. (2022);** **Arkell et al. (2022);** **Arkell et al. (2020)**	DB, PC, Ra, Cr		26	23.2 (2.6)	10/16	**Bedrocan** (13.75 mg THC) **Bedrocan + Bedrolite** (13.75 mg THC +13.75 mg CBD) **Bedrolite** (13.75 mg CBD)	Vapor inhalation	Single dose	Blood (plasma)	THC 11-OH-THC 11-COOH-THC CBD	[3.75 mg THC] THC: 22.91 (12.58) CBD: 0.02 (0.07) [13.75 mg THC +13.75 CBD] THC: 19.98 (3.36) CBD: 13.92 (8.47) [13.75 mg CBD] THC: 1.75 (2.13) CBD: 15.82 (6.92)	Direct after administration
**Oliver et al. (2023);** **Englund et al. (2023);** **Chester et al. (2022)**	DB, Ra, Cr		46	26.62 (4.94)	25/21	**Bedrocan** (10 mg THC) **Bedrocan + Bedrolite** (10 mg THC +10 mg CBD) **Bedrocan + Bedrolite** (10 mg THC +20 mg CBD) **Bedrocan + Bedrolite** (10 mg THC +30 mg CBD)	Vapor inhalation	Single dose	Blood (plasma)	THC COOH-THC OH–THC CBD 7-OH-THC AEA 2-AG ARA-S DEA OEA SEA aLEA gLEA	[10 mg THC] THC: 34.54 (21.64) CBD: 0.04 (0.28) [10 mg THC +10 mg CBD] THC: 36.8 (19.1) CBD: 30.12 (17.19) [10 mg THC +20 mg CBD] THC: 39.26 (26.76) CBD: 67.43 (50.16) [10 mg THC +30 mg CBD] THC: 38.46 (23.14) CBD: 96.12 (64.25)	5
**de Bruijn et al. (2017)**	DB, PC, Ra, Cr		10	23.4 (1)	10/0	**Bedrocan** (4 mg THC) + 1 mg THC) **Bedrolite** (25 mg CBD) + 10 mg CBD)	Vapor inhalation	Single dose + top-up dose after 35 min	Blood (plasma)	THC CBD	[4 mg THC +1 mg THC] THC: 1.6 (0.5, SEM) CBD: 0.34 (0.1, SEM) [25 mg CBD +10 mg CBD] THC: 0.16 CBD: 2.8 (0.8, SEM)	30
**Hunault et al. (2008);** **Hunault et al. (2014)**	DB, PC, Ra, Cr		24	23.9 (4.1)	24/0	**Bedrocan** (69.4 mg THC) **Bedrocan** (49.1 mg THC) **Bedrocan** (29.3 mg THC)	Smoking	Single dose	Blood (serum)	THC THC–COOH 11-OH–THC	[29.3 mg THC] THC: 135.1 (68.5) [49.1 mg THC] THC: 202.9 (112.4) [69.4 mg THC] THC: 231.0 (108.5)	5
**Lawn et al. (2023);** **Skumlien et al. (2023)**	DB, PC, Ra, Cr	Adolescents Adults	24 24 **48**	17.17 (0.43) 27.77 (1.04)	12/12 12/12	**Bedrocan** (0.107 mg/kg THC) **Bedrocan + Bedrolite** (0.107 mg/kg THC +0.320 mg/kg CBD)	Vapor inhalation	Single dose	Blood (plasma)	THC CBD	[0.107 mg/kg THC; Adolescents] THC: 13.67 (6.74) CBD: 0 (0) [0.107 mg/kg THC; Adults] THC: 16.10 (7.48) CBD: 0.16 (0.54) [0.107 mg/kg THC +0.320 mg/kg CBD; Adolescents] THC: 24.74 (9.00) CBD: 44.32 (15.33) [0.107 mg/kg THC +0.320 mg/kg CBD; Adults] THC: 30.84 (17.51) CBD: 65.34 (34.84)	20
**Mason et al. (2019)**	DB, PC, Ra, Cr	Full dose Successive doses	10 10 **20**	22.5 (0.86) 21.2 (0.85)	12/8	**Bedrobinol** (full dose: 0.3 mg/kg THC) **Bedrobinol** (successive doses: 3 times 0.1 mg/kg THC)	Vapor inhalation	Single dose or 3 successive doses (separated by 30 min)	Blood (serum)	THC THC–COOH OH–THC	[Full dose] THC: 7.03 (1.83, SE) [Successive dose] THC: 1.66 (0.49, SE)	6
**Mason et al. (2021)**	DB, PC, Ra, Cr	Occasional users Chronic users	12 12 **24**	22.5 (2.54) 21.83 (2.25)	5/7 9/3 **14/10**	**Bedrobinol** (0.3 mg/kg THC)	Vapor inhalation	Single dose	Blood (serum)	THC THC–COOH OH–THC	[Occasional users] THC: 7.80 (1.69, SE) [Chronic users] THC: 15.86 (3.48, SE)	6
**Theunissen et al. (2015) ^b^**	DB, PC, Ra, Cr		15	21.23 (1.76)	9/6	Placebo + **Bedrobinol** (0.15 mg/kg THC) Vardenafil (20 mg) + **Bedrobinol** (0.15 mg/kg THC) Rivastigmine (3 mg) + **Bedrobinol** (0.15 mg/kg THC)	Vapor inhalation	Single dose	Blood (serum)	THC THC–COOH OH–THC	[Placebo +0.15 mg/kg THC] THC: 30.7 (27.4)	Direct after administration
**Battistella et al. (2013)**	DB, PC, Ra, Cr		31	24.1 (3)	31/0	**Bedrobinol** (42 mg THC)	Smoking	Single dose	Blood (whole blood)	THC THC–COOH 11-OH–THC	THC: median value of 87.4 (range: 16.8–167.9)	Direct after administration
**Fabritius et al. (2013)**	DB, PC, Ra, Cr	Heavy users Occasional users	23 25 **48**	22.7 (2.4) 23.9 (3.0)	23/0 25/0 **48/0**	**Bedrobinol** (42 mg THC)	Smoking	Single dose	Blood (whole blood) Saliva	THC THCCOOH 11-OH-THC THC-A CBD CBN	[Blood; Heavy users] THC: 95 (47) [Blood; Occasional users] THC: 76 (46) [Saliva; Heavy users] THC: 1047 (967) [Saliva; Occasional users] THC: 1388 (782)	Blood: 20 Saliva: 20–30
**Brenneisen et al. (2010)**	OL		12	26 (3)	12/0	**Bedrobinol** (70 mg THC)	Smoking	Single dose	Blood (plasma) Urine	TH THC–COOH THC-OH	[Plasma] THC: 20.9 (16.9) [Urine] THC: 0.2 (1.3)	Blood: 5 Urine: 120
**Ramaekers et al. (2006a);** **Ramaekers et al. (2006b)**	DB, PC, Ra, Cr		20	19–29 years	14/6	**Bedrobinol** (0.5 mg/kg THC) **Bedrobinol** (0.25 mg/kg THC)	Smoking	Single dose	Blood (serum) Saliva	THC THC–COOH OH-THC	[Blood; 0.5 mg/kg THC] THC: 95.1 (63.2) [Blood; 0.25 mg/kg THC] THC: 58.0 (47.7) [Saliva; 0.5 mg/kg THC] THC: 918 (702) [Saliva; 0.25 mg/kg THC] THC: 899 (630)	Blood: 5 Saliva: 15
**Ramaekers et al. (2009)**	DB, PC, Ra, Cr	Occasional users Heavy users	12 12 **24**	22.8 (2.3) 23.2 (3.3)	8/4 9/3 **17/1**	**Bedrobinol** (0.5 mg/kg THC)	Smoking	Single dose	Blood (serum)	THC THC–COOH OH-THC	[Occasional users] THC: 49.1 (24.9) [Heavy users] THC: 120.9 (78.1)	5
**Ramaekers et al. (2011)**	DB, PC, Ra, Cr		19	23.2 (8.4)	15/6	**Bedrobinol** (0.4 mg/kg THC) + 700 mg/kg alcohol **Bedrobinol** (0.4 mg/kg THC) + 500 mg/kg alcohol **Bedrobinol** (0.4 mg/kg THC)	Smoking	Single dose	Blood (serum)	THC THC–COOH OH-THC	[0.4 mg/kg THC] THC: 112.1 (47.5)	15
**Ramaekers et al. (2022)**	DB, PC, Ra, Cr	Occasional users Chronic users	14 12 **26**	22.14 (2.51) 21.83 (2.25)	7/7 9/3 **16/10**	**Bedrobinol** (0.3 mg/kg THC)	Vapor inhalation	Single dose	Blood (serum)	THC THC–COOH THC-OH	[Occasional users mean] THC: 8.61 (1.46, SE) [Chronic users] THC: 15.86 (3.48, SE)	6
**Spronk et al. (2016a)^b^**	DB, PC, Ra, Cr		61	22.6 (4.3)	12/49	**Bedrobinol** (0.3 mg of THC/kg) + 0.15 mg of THC/kg)	Vapor inhalation	Single dose + second dose after 1 h	Blood (serum)	THC THC–COOH THC-OH	THC: 89.09 (80.6)	5
**Spronk et al. (2016a)^b^**	DB, PC, Ra, Cr		38	22.1 (4.6)	9/29	**Bedrobinol** (0.3 mg of THC/kg) + 0.15 mg of THC/kg)	Vapor inhalation	Single dose + second dose after 1 h	Blood (serum)	THC THC–COOH THC-OH	THC: 117.22 (98.71)	5
**Tank et al. (2019)**	OL		15	25	3/12	**Bedrocan** (0.3 mg/kg THC)	Smoking	Single dose	Blood (serum)	THC THC–COOH 11-OH–THC	THC: ranged from 2.4 to 42.9	Direct after administration

*Combination treatment with medicinal cannabis from different companies, only Bedrocan products and dosing are reported.

^a^ In bold: cannabis variety; regular: mg THC/CBD.

^b^ Only conditions that include cannabis are displayed. DB, double blind; PC, placebo controlled; Ra, Randomized; Cr, Crossover; OL, Open-Label.

**Table 2:** Overview of studies investigating subjective effects after cannabis administration.

First author	Study design	Groups (if applicable)	N	Mean age (SD)	M/F	Study medication^b^	Route	Frequency	Main effects (group/condition significant effect)
**Battistella et al. (2013)**	DB, PC, Ra, Cr		31	24.1 (3)	31/0	**Bedrobinol** (42 mg THC)	Smoking	Single dose	↑ Intoxication ↑ Confusion ↑ High ↑ Change in the environment ↓ Ability to drive
**Arkell et al. (2020)**	DB, PC, Ra, Cr		26	23.2 (2.6)	10/16	**Bedrocan** (13.75 mg THC) **Bedrocan + Bedrolite** (13.75 mg THC +13.75 mg CBD) **Bedrolite** (13.75 mg CBD)	Vapor inhalation	Single dose	↑ Drug effect = Liking ↑ Stoned (THC + THC/CBD)↑ Sedated (THC + THC/CBD) ↓ Relaxed (THC)↑ Anxious (THC + THC/CBD)↓ Confident to drive (THC + THC/CBD)
**Arkell et al. (2022)^a^**	DB, PC, Ra, Cr	Male Female	21 19 **40**	25.8 (3.6) 23.5 (4.0)	19/21	**Bedrocan** (13.75 mg THC) **Bedrocan + Bedrolite** (13.75 mg THC +13.75 mg CBD) **Bedrolite** (13.75 mg CBD)	Vapor inhalation	Single dose	↑ Strength of drug effect ↑ Liking of drug effect ↑ Stoned ↑ Sedated ↑ Anxious ↓ Confident to drive
**Hunault et al. (2008);** **Hunault et al. (2009);** **Hunault et al. (2014)**	DB, PC, Ra, Cr		24	23.9 (4.1)	24/0	**Bedrocan** (69.4 mg THC) **Bedrocan** (49.1 mg THC) **Bedrocan** (29.3 mg THC)	Smoking	Single dose	↑ Feeling high, (increased with dose) ↑ Drowsiness ↓ Capability to perform a task (decreased with dose) ↓ Alert ↓ Content ↓ Calm ↑ Dizziness ↑ Dry mouth ↑ Papilation ↑ Impaired memory ↑ Down ↑ Sedated ↑ Anxious
**Fabritius et al. (2013)**	DB, PC, Ra, Cr	Heavy users Occasional users	23 25 **48**	22.7 (2.4) 23.9 (3.0)	23/0 25/0 **48/0**	**Bedrobinol** (42 mg THC)	Smoking	Single dose	↑ Intoxication ↑ Confusion (heavy users)
**Theunissen et al. (2015) ^c^**	DB, PC, Ra, Cr		15	21.23 (1.76)	9/6	Placebo + **Bedrobinol** (0.15 mg/kg THC) Vardenafil (20 mg) + **Bedrobinol** (0.15 mg/kg THC) Rivastigmine (3 mg) + **Bedrobinol** (0.15 mg/kg THC)	Vapor inhalation	Single dose	↑ High
**Mason et al. (2019)**	DB, PC, Ra, Cr	Full dose Successive doses	10 10 **20**	22.5 (0.86) 21.2 (0.85)	12/8	**Bedrobinol** (full dose: 0.3 mg/kg THC) **Bedrobinol** (successive doses: 3 times 0.1 mg/kg THC)	Vapor inhalation	Single dose or 3 successive doses (separated by 30 min)	↑ High (full-dose)
**Ramaekers et al. (2022)**	DB, PC, Ra, Cr	Occasional users Chronic users	14 12 **26**	22.14 (2.51) 21.83 (2.25)	7/7 9/3 **16/10**	**Bedrobinol** (0.3 mg/kg THC)	Vapor inhalation	Single dose	↑ High
**Ramaekers et al. (2006a)**	DB, PC, Ra, Cr		20	19–29 years	14/6	**Bedrobinol** (0.5 mg/kg THC) **Bedrobinol** (0.25 mg/kg THC)	Smoking	Single dose	↑ High
**Ramaekers et al. (2009)**	DB, PC, Ra, Cr	Occasional users Heavy users	12 12 **24**	22.8 (2.3) 23.2 (3.3)	8/4 9/3 **17/1**	**Bedrobinol** (0.5 mg/kg THC)	Smoking	Single dose	↑ High
**Ramaekers et al. (2011)**	DB, PC, Ra, Cr		19	23.2 (8.4)	15/6	**Bedrobinol** (0.4 mg/kg THC) + 700 mg/kg alcohol **Bedrobinol** (0.4 mg/kg THC) + 500 mg/kg alcohol **Bedrobinol** (0.4 mg/kg THC)	Smoking	Single dose	↑ High
**Kowal et al. (2015a);** **Kowal et al. (2015b)**	DB, PC, Ra, BS	Placebo 5.5 mg THC 22 mg THC	19 18 18 **55**	21.3 (2.3) 21.1 (2.1) 22.3 (2.3)	18/1 17/1 14/4	**Bedrocan** (22 mg THC) **Bedrocan** (5.5 mg THC)	Vapor inhalation	Single dose	↑ High ↑ Good drug effect ↑ Bad drug effect (22 mg THC)
**Spronk et al. (2016a)^c^; Spronk et al. (2016b)^c^; Spronk et al. (2016c)^c^**	DB, PC, Ra, Cr		61	22.6 (4.3)	49/12	**Bedrobinol** (0.3 mg of THC/kg) + 0.15 mg of THC/kg)	Vapor inhalation	Single dose + second dose after 1 h	↑ High ↓ Feeling active
**Lawn et al. (2016);** **Wall et al. (2022);** **Mokrysz et al. (2021)**	DB, PC, Ra, Cr		17	26.18 (7.13)	8/9	**Bediol** (8 mg THC) **Bedrobinol** (8 mg THC +10 mg CBD)	Vapor inhalation	Single dose +50% top-up dose after 90 min	↑ Stoned
**Mokrysz et al. (2016); Mokrysz et al. (2021)**	DB, PC, Ra, Cr	Adolescents Adults	20 20 **40**	17.08 (0.44) 25.49 (1.07)	40/0	**Bedrobinol** (0.107 mg/kg THC)	Vapor inhalation	Single dose	↑ Stoned (higher in adults) ↑ High ↑ Feel drug effect (higher in adults) ↑ Like drug effect ↓ Alert (adults) ↑ Anxious (adults) ↑ Paranoid ↑ Dry mouth (higher in adults) ↑ Enhanced colour perception ↑ Enhanced sound perception ↑ Want to have food ↑ Want to have cannabis (adolescent; ↓ adults) ↑ Mentally impaired
**Mason et al. (2021)**	DB, PC, Ra, Cr	Occasional users Chronic users	12 12 **24**	22.5 (2.54) 21.83 (2.25)	5/7 9/3 **14/10**	**Bedrobinol** (0.3 mg/kg THC)	Vapor inhalation	Single dose	↑ High (higher in occasional users)
**Lawn et al. (2023)**	DB, PC, Ra, Cr	Adolescents Adults	24 24 **48**	17.17 (0.43) 27.77 (1.04)	12/12 12/12	**Bedrocan** (0.107 mg/kg THC) **Bedrocan + Bedrolite** (0.107 mg/kg THC +0.320 mg/kg CBD)	Vapor inhalation	Single dose	↑ Feeling drug effect (THC) ↑ Anxious (THC) ↑ Paranoid (THC) = Want cannabis ↑ Like drug effect (THC) = Dislike drug effect ↑ Happy (adolescent; ↓ adults)
**de Bruijn et al. (2017)**	DB, PC, Ra, Cr		10	23.4 (1)	10/0	**Bedrocan** (4 mg THC) + 1 mg THC) **Bedrolite** (25 mg CBD) + 10 mg CBD)	Vapor inhalation	Single dose + top-up dose after 35 min	↑ Feeling high (THC)
**van Dam et al. (2023)**	DB, PC, Ra, Cr		18	22 (3)	9/9	Placebo + **Bedrocan** (21.8 mg THC) Oxycodone (20 mg) + **Bedrocan** (21.8 mg THC)	Vapor inhalation	Single dose	↑ Sedation (higher in THC + Oxycodone) ↑ Drug high ↑ Drowsy (higher in THC + Oxycodone) ↓ Energetic (higher in THC + Oxycodone)
**Freeman et al. (2021)^a^**	DB, PC, Ra, Cr		128	22.66 (4.41)	35/93	**Bediol** (8 mg THC)	vapor inhalation	Single dose	↓ Alertness ↑ Stoned = Anxiety = Wanting more cannabis

^a^ Combination treatment with medicinal cannabis from different companies, only Bedrocan products and dosing are reported.

^b^ In bold: cannabis variety; regular: mg THC/CBD.

^c^ only conditions that include cannabis are displayed. DB, double blind; PC, placebo controlled; Ra, Randomized; Cr, Crossover.

**Table 3:** Overview of studies investigating cognitive effects after cannabis administration.

First author	Study design	Groups (if applicable)	N	Mean age (SD)	M/F	Study medication^b^	Route	Frequency	Main effects (group/condition significant effect)
**Arkell et al. (2020)**	DB, PC, Ra, Cr		26	23.2 (2.6)	10/16	**Bedrocan** (13.75 mg THC) **Bedrocan + Bedrolite** (13.75 mg THC +13.75 mg CBD) **Bedrolite** (13.75 mg CBD)	Vapor inhalation	Single dose	Processing speed [Digit Symbol Substitution Task]↓ number correct (THC and THC/CBD) Divided attention [Divided Attention Task]↑ response time (THC and THC/CBD)↓ number correct (THC and THC/CBD) Working memory [Paced Serial Addition Task]↑ response time (THC and THC/CBD)↓ number correct (THC and THC/CBD) Forward planning [Tower of London] ↓ response time (THC) Standard deviation of lateral position (a measure of lane weaving) ↑ (THC and THC/CBD)
**Englund et al. (2023)**	DB, Ra, Cr		46	26.62 (4.94)	25/21	**Bedrocan** (10 mg THC) **Bedrocan + Bedrolite** (10 mg THC +10 mg CBD) **Bedrocan + Bedrolite** (10 mg THC +20 mg CBD) **Bedrocan + Bedrolite** (10 mg THC +30 mg CBD)	Vapor inhalation	Single dose	Learning and memory [Hopkins verbal learning task] ↓ immediate and delayed recall (compared to baseline) ↑ intrusions (compared to baseline) Verbal working memory and attention [Digit span] ↓ forward digit span (compared to baseline) = reverse digit span (compared to baseline) Memory [Spatial N-Back] = 0–2 back (compared to baseline)
**Oliver et al. (2023)**	DB, Ra, Cr		46	26.62 (4.94)	25/21	**Bedrocan** (10 mg THC) **Bedrocan + Bedrolite** (10 mg THC +10 mg CBD) **Bedrocan + Bedrolite** (10 mg THC +20 mg CBD) **Bedrocan + Bedrolite** (10 mg THC +30 mg CBD)	Vapor inhalation	Single dose	Attentional bias [Attentional bias task] = bias towards neutral stimuli/food/cannabis
**Hunault et al. (2009)**	DB, PC, Ra, Cr		23	24.1 (4)	23/0	**Bedrocan** (69.4 mg THC) **Bedrocan** (49.1 mg THC) **Bedrocan** (29.3 mg THC)	Smoking	Single dose	Reaction time [Simple reaction time test] ↑ response time (THC dose effect) Visuo-spatial selective attention [Erickson flanker task] ↑ response time (THC dose effect) Short-term memory [Sternberg’s memory scanning test] ↑ response time (THC dose effect) ↑ number of errors (THC dose effect) Moto control [Motor control task] ↑ tracking deviation (THC dose effect) Divided attention [Divided attention task] ↑ response time (THC dose effect) Sustained attention [Sustained attention task] ↑ response time (THC dose effect) ↑ number of errors (THC dose effect)
**Kowal et al. (2015a)**	DB, PC, Ra, BS	Placebo 5.5 mg THC 22 mg THC	19 18 18 **55**	21.3 (2.3) 21.1 (2.1) 22.3 (2.3)	1/18 1/17 4/14	**Bedrocan** (22 mg THC) **Bedrocan** (5.5 mg THC)	Vapor inhalation	Single dose	Error monitoring [Flanker task] ↑ omissions (22 mg THC) = reaction time = post-error slowing
**Kowal et al. (2015b)**	DB, PC, Ra, BS	Placebo 5.5 mg THC 22 mg THC	18 18 18 **54**	21.1 (2.4) 21.1 (2.1) 22.0 (2.5)	0/18 1/17 5/13	**Bedrocan** (22 mg THC) **Bedrocan** (5.5 mg THC)	Vapor inhalation	Single dose	Divergent thinking [Alternate Uses Task] ↓ fluency (22 mg THC) ↓ flexibility (22 mg THC) ↓ originality (22 mg THC) = elaboration Convergent thinking [Remote Associates Task] = number of correct items
**Lawn et al. (2016)** **S*tudy 1***	DB, PC, Ra, Cr		17	26.18 (7.13)	8/9	**Bediol** (8 mg THC) **Bedrobinol** (8 mg THC +10 mg CBD)	Vapor inhalation	Single dose +50% top-up dose after 90 min	Effort related decision making [Effort expenditure for rewards task] ↓ high-effort choice ↑ sensitivity to expected value (THC)
**Lawn et al. (2023)**	DB, PC, Ra, Cr	Adolescents Adults	24 24 **48**	17.17 (0.43) 27.77 (1.04)	12/12 12/12	**Bedrocan** (0.107 mg/kg THC) **Bedrocan + Bedrolite** (0.107 mg/kg THC +0.320 mg/kg CBD)	Vapor inhalation	Single dose	Verbal episodic memory [Delayed prose recall] ↓ recall
**Mokrysz et al. (2016)**	DB, PC, Ra, Cr	Adolescents Adults	20 20 **40**	17.08 (0.44) 25.49 (1.07)	40/0	**Bedrobinol** (0.107 mg/kg THC)	Vapor inhalation	Single dose	Memory [Prose recall] ↓ recalled items Memory [Spatial N-back] ↑ reaction time (adults) Response inhibition [Stop signal] ↓ accuracy (adolescents)
**Mason et al. (2019)**	DB, PC, Ra, Cr	Full dose Successive doses	10 10 **20**	22.5 (0.86) 21.2 (0.85)	12/8	**Bedrobinol** (full dose: 0.3 mg/kg THC) **Bedrobinol** (successive doses: 3 times 0.1 mg/kg THC)	Vapor inhalation	Single dose or 3 successive doses (separated by 30 min)	Sustained attention [Psychomotor vigilance task] = mean reaction time ↑ number of attentional lapses (full dose)
**Mason et al. (2021)**	DB, PC, Ra, Cr	Occasional users Chronic users	12 12 **24**	22.5 (2.54) 21.83 (2.25)	5/7 9/3 **14/10**	**Bedrobinol** (0.3 mg/kg THC)	Vapor inhalation	Single dose	Sustained attention [Psychomotor vigilance task] ↑ mean reaction time (occasional users) ↑ number of attentional lapses (occasional users)
**Battistella et al. (2013)**	DB, PC, Ra, Cr		31	24.1 (3)	31/0	**Bedrobinol** (42 mg THC)	Smoking	Single dose	Perceptual-motor control [Critical tracking task] ↓ tracking skills
**Ramaekers et al. (2006a);** **Ramaekers et al. (2006b)**	DB, PC, Ra, Cr		20	19–29 years	14/6	**Bedrobinol** (0.5 mg/kg THC) **Bedrobinol** (0.25 mg/kg THC)	Smoking	Single dose	Perceptual motor control [Critical tracking task] ↓ lambda-c Motor impulsivity [Stop signal task] ↑ reaction time (0.5 mg THC) ↑ number of omission errors Cognitive function [Tower of London] ↓ number of correct decisions = planning time Decision making [Iowa gambling task] = advantageous/disadvantageous choices
**Ramaekers et al. (2009)**	DB, PC, Ra, Cr	Occasional users Heavy users	12 12 **24**	22.8 (2.3) 23.2 (3.3)	8/4 9/3 **17/1**	**Bedrobinol** (0.5 mg/kg THC)	Smoking	Single dose	Perceptual motor control [Critical tracking task] ↑ lambda-c (occasional users) Dual task processing [divided-attention task] ↑ tracking error (occasional users) ↑ number of losses (occasional users) ↓ number of hits (occasional users) Motor inhibition [Stop-signal task] ↑ reaction time ↓ accuracy of responses = reaction time on go-trials Cognition [Tower of London] = number of correct decisions = mean reaction time
**Ramaekers et al. (2011)**	DB, PC, Ra, Cr		19	23.2 (8.4)	15/6	**Bedrobinol** (0.4 mg/kg THC) + 700 mg/kg alcohol **Bedrobinol** (0.4 mg/kg THC) + 500 mg/kg alcohol **Bedrobinol** (0.4 mg/kg THC)	Smoking	Single dose	Perceptual motor control [Critical tracking task] = lambda-c Dual task processing [divided-attention task] ↑ number of control losses ↑ reaction time ↓ number of correct signal detections Motor inhibition [Stop-signal task] = stop reaction time = commission errors Cognition [Tower of London] = number of correct decisions = mean reaction time
**Ramaekers et al. (2022)**	DB, PC, Ra, Cr	Occasional users Chronic users	14 12 **26**	22.14 (2.51) 21.83 (2.25)	7/7 9/3 **16/10**	**Bedrobinol** (0.3 mg/kg THC)	Vapor inhalation	Single dose	Sustained attention [Psychomotor vigilance task] ↑ number of attentional lapses (occasional users)
**Spronk et al. (2016a)^c^**	DB, PC, Ra, Cr		61	22.6 (4.3)	12/49	**Bedrobinol** (0.3 mg of THC/kg) + 0.15 mg of THC/kg)	Vapor inhalation	Single dose + second dose after 1 h	Learning [Reversal learning paradigm] ↓ accuracy ↑ reaction time Switch attention [Attention switch task] ↑ errors Forward planning [Tower of London] ↓ portion correct ↑ reaction time
**Spronk et al. (2016a)^c^**	DB, PC, Ra, Cr		61	22.6 (4.3)	12/49	**Bedrobinol** (0.3 mg of THC/kg) + 0.15 mg of THC/kg)	Vapor inhalation	Single dose + second dose after 1 h	Performance monitoring [Flanker task] = error rate ↑ reaction time
**Spronk et al. (2016b)^c^**	DB, PC, Ra, Cr		38	22.1 (4.6)	9/29	**Bedrobinol** (0.3 mg of THC/kg) + 0.15 mg of THC/kg)	Vapor inhalation	Single dose + second dose after 1 h	Response inhibition [Go/NoGo task] ↑ commission errors ↑ reaction time
**Kloft et al. (2020)**	DB, PC, Ra, Cr		64	22.7 (2.6)	32/32	**Bedrobinol** (0.3 mg of THC/kg)	Vapor inhalation	Single dose	False memory [associative word lists and two misinformation tasks] ↑ false memory rates = true memory
**Freeman et al. (2021)^a^**	DB, PC, Ra, Cr		128	22.66 (4.41)	35/93	**Bediol** (8 mg THC)	Vapor inhalation	Single dose	Episodic memory [Prose recall task] ↓ number of units recalled

^a^ Combination treatment with medicinal cannabis from different companies, only Bedrocan products and dosing are reported.

^b^ In bold: cannabis variety; regular: mg THC/CBD.

^c^ only conditions that include cannabis are displayed. DB, double blind; PC, placebo controlled; Ra, Randomized; Cr, Crossover; BS, between subjects.

**Table 4:** Overview of studies investigating the effects of cannabis CBD/THC ratio.

First author	Study design	Groups (if applicable)	N	Mean age (SD)	M/F	Study Medication^a^	Route	Frequency	Main effects THC vs. CBD/THC (group/condition significant effect)
**Arkell et al. (2020)**	DB, PC, Ra, Cr		26	23.2 (2.6)	10/16	**Bedrocan** (13.75 mg THC) **Bedrocan + Bedrolite** (13.75 mg THC +13.75 CBD) **Bedrolite** (13.75 mg CBD)	Vapor inhalation	Single dose	Standard deviation of lateral position (a measure of lane weaving): =Confident to drive: ↑ THC/CBD Strength of drug effect: ↑ THCAnxious: ↓ THC/CBD
**Hutten et al. (2022)**	DB, PC, Ra, Cr		26	23.1 (2.6)	10/16	**Bedrocan** (13.75 mg THC) **Bedrocan + Bedrolite** (13.75 mg THC +13.75 CBD) **Bedrolite** (13.75 mg CBD)	Vapor inhalation	Single dose	State anxiety: ↓ THC/CBDSubjective anxiety: ↓ THC/CBD Emotional Stroop: =
**Lawn et al. (2016)** **S*tudy 1***	DB, PC, Ra, Cr		17	26.18 (7.13)	8/9	**Bediol** (8 mg THC) **Bedrobinol** (8 mg THC +10 mg CBD)	Vapor inhalation	Single dose +50% top-up dose after 90 min	Stoned rating: = Effort related decision making task High-effort choice: = Sensitivity to expected value: ↑ THC
**Wall et al. (2019)**	DB, PC, Ra, Cr		17	26.2 (7.1)	8/9	**Bediol** (8 mg THC) **Bedrobinol** (8 mg THC +10 mg CBD)	Vapor inhalation	Single dose	Brain resting state networks - connectivity Default mode network: = Executive control network: = Salience network: ↓ THC
**Wall et al. (2022)** ***Study 1***	DB, PC, Ra, Cr		17	26.2 (7.1)	8/9	**Bediol** (8 mg THC) **Bedrobinol** (8 mg THC +10 mg CBD)	Vapor inhalation	Single dose	Functional connectivity of striatal sub-divisions Associative: = Limbic: ↓ THC Sensorimotor: =
**Mokrysz et al. (2021)** ***Study 1***	DB, PC, Ra, Cr		17	24 (4.5)	8/9	**Bediol** (8 mg THC) **Bedrobinol** (8 mg THC +10 mg CBD)	Vapor inhalation	Single dose +50% top-up dose after 90 min	Speech illusion: = Psychotic-like symptoms: =
**Chester et al. (2022)**	DB, Ra, Cr		46	26.62 (4.94)	25/21	**Bedrocan** (10 mg THC) **Bedrocan + Bedrolite** (10 mg THC + 10 mg CBD) **Bedrocan + Bedrolite** (10 mg THC +20 mg CBD) **Bedrocan + Bedrolite** (10 mg THC +30 mg CBD)	Vapor inhalation	Single dose	Plasma THC: = concentration, peak and AUC Plasma CBD: ↑ dose-dependent increase in peak and AUC CBD:THC ratios: = peak or AUC plasma concentrations for any of the endocannabinoids or related noncannabinoid lipids
**Englund et al. (2023)**	DB, Ra, Cr		46	26.62 (4.94)	25/21	**Bedrocan** (10 mg THC) **Bedrocan + Bedrolite** (10 mg THC +10 mg CBD) **Bedrocan + Bedrolite** (10 mg THC +20 mg CBD) **Bedrocan + Bedrolite** (10 mg THC +30 mg CBD)	Vapor inhalation	Single dose	Hopkins verbal learning task: = Digit span: = Spatial N-Back: = Psychotic symptoms: =
**Oliver et al. (2023)**	DB, Ra, Cr		46	26.62 (4.94)	25/21	**Bedrocan** (10 mg THC) **Bedrocan + Bedrolite** (10 mg THC +10 mg CBD) **Bedrocan + Bedrolite** (10 mg THC +20 mg CBD) **Bedrocan + Bedrolite** (10 mg THC +30 mg CBD)	Vapor inhalation	Single dose	Attentional bias: = Explicit liking: =
**Skumlien et al. (2023)**	DB, PC, Ra, Cr	Adolescents Adults	24 23 **47**	17.17 (0.43) 27.8 (1.06)	12/12 12/11	**Bedrocan** (0.107 mg/kg THC) **Bedrolite** (0.107 mg/kg THC +0.320 mg/kg CBD)	Vapor inhalation	Single dose	Reward anticipation activity Right ventral striatum: = Left ventral striatum: = Right anterior cingulate cortex: = Left anterior cingulate cortex: = Right insula: =
**Lawn et al. (2023)**	DB, PC, Ra, Cr	Adolescents Adults	24 24 **48**	17.17 (0.43) 27.77 (1.04)	12/12 12/12	**Bedrocan** (0.107 mg/kg THC) **Bedrolite** (0.107 mg/kg THC +0.320 mg/kg CBD)	Vapor inhalation	Single dose	Subjective drug effect: = Verbal episodic memory: = Psychotomimetic effect: =

^a^ In bold: cannabis variety; regular: mg THC/CBD; DB, double blind; PC, placebo controlled; Ra, Randomized; Cr, Crossover.

#### 3.1.1 Pharmacokinetics of THC and CBD

25 papers reported data on the pharmacokinetics of THC, CBD, and/or their metabolites after smoking or inhalation of standardized cannabis (Table 1). Although drug delivery through smoking and inhalation is comparable in terms of drug absorption, smoking is highly discouraged by healthcare professionals because of its harmful effects (Chaiton et al., 2022). The main reason that smoking is used as route of administration in studies with healthy volunteers is that it is the preferred consumption method for recreational cannabis use (Grotenhermen, 2003; United Nations Office on Drugs and Labor, 2021). THC concentrations were reported in all papers. Peak THC concentrations measured in blood (serum, plasma, and whole blood) were shown directly after and up to 30 min following cannabis administration (depending on the timing of blood sampling). However, substantial inter-individual variability in these concentrations was observed. Two studies administered cannabis with different doses of THC (Bedrocan^®^ or Bedrobinol^®^) and demonstrated a dose-dependent increase in THC concentrations in blood (Hunault et al., 2008; Hunault et al., 2014; Ramaekers et al., 2006a; Ramaekers et al., 2006b). Four studies also administered CBD-dominant cannabis or cannabis containing a combination of both THC and CBD (Arkell et al., 2020; Chester et al., 2022; de Bruijn et al., 2017; Englund et al., 2023; Hutten et al., 2022; Lawn et al., 2023; Oliver et al., 2023; Skumlien et al., 2023) and demonstrated a dose-dependent increase in CBD plasma concentrations. Finally, four studies investigated the impact of cannabis use experience on THC concentrations (Fabritius et al., 2013; Mason et al., 2021; Ramaekers et al., 2009; Ramaekers et al., 2022) and showed that inhaling an equal dose of Bedrobinol^®^ resulted in significantly higher THC concentrations in chronic compared to occasional cannabis users. Altogether, kinetic findings indicate a dose-dependent increase in THC and CBD blood levels shortly after standardized cannabis administration (smoking or vaporizing) which may be influenced by cannabis use history.

#### 3.1.2 Acute subjective experiences

29 papers (consisting of 21 unique studies) reported on subjective experiences after administration (smoking or vaporizing) of standardized cannabis to healthy volunteers in comparison to placebo, see Table 2. All studies reported acute subjective effects of feeling stoned, high, or intoxicated, which were present within 5 min and peaked at or shortly after standardized cannabis administration. Effects appeared to be dose-dependent, with both stronger effects of feeling high and unwanted drug effects after cannabis with higher doses of THC (Hunault et al., 2008; Hunault et al., 2009; Hunault et al., 2014; Kowal et al., 2015a; Kowal et al., 2015b). Six studies also assessed subjective anxiety (Arkell et al., 2020; Freeman et al., 2021; Hunault et al., 2008; Hunault et al., 2009; Hunault et al., 2014; Lawn et al., 2023; Mokrysz et al., 2016; Mokrysz et al., 2021) and five of these studies reported increased feelings of anxiety after THC-containing cannabis administration (either THC-dominant cannabis or cannabis with THC and CBD combined) (Arkell et al., 2020; Hunault et al., 2008; Hunault et al., 2009; Hunault et al., 2014; Lawn et al., 2023; Mokrysz et al., 2016; Mokrysz et al., 2021). Subjective ratings of anxiety after cannabis (Arkell et al., 2020; Lawn et al., 2023) were more pronounced in adults than in adolescents (Mokrysz et al., 2016; 2021). Other acute subjective experiences that were reported after THC-containing cannabis included feelings of increased sedation, confusion, paranoia and diminished alertness and energy (Arkell et al., 2020; Battistella et al., 2013; Fabritius et al., 2013; Freeman et al., 2021; Hunault et al., 2008; 2009; 2014; Mokrysz et al., 2016; Mokrysz et al., 2021; Spronk et al., 2016b; Spronk et al., 2016b; Spronk et al., 2016c; van Dam et al., 2023. Administration of CBD-dominant cannabis did not induce any subjective effects including feeling high and anxiety (Arkell et al., 2020; de Bruijn et al., 2017; Hutten et al., 2022). Altogether, these studies suggest dose-dependent subjective cannabis effects of feeling high or stoned, which are present shortly after administration of THC-containing cannabis. In addition, more negative drug effects are also reported, such as feelings of anxiety, confusion, and paranoia. CBD-dominant cannabis does not seem to induce acute subjective effects.

#### 3.1.3 Impact on cognitive assessments

22 papers (consisting of 21 unique studies) reported on cognitive effects after administration (smoking or vaporizing) of standardized cannabis in comparison to placebo, see also Table 3. First, 21 cognitive tasks measuring for example, attention, processing speed, and memory reported on changes in reaction time. On most of these cognitive tasks (16 out of 21), THC-containing cannabis resulted in a significant delay in response time (Arkell et al., 2020; Hunault et al., 2009; Ramaekers et al., 2009; 2011; Ramaekers et al., 2006a; Ramaekers et al., 2006b; Spronk et al., 2016a; Spronk et al., 2016b; Spronk et al., 2016c). In 11 of the 12 tasks that also included a measure of performance accuracy, it was demonstrated that the delay in response time after THC-containing cannabis was accompanied by impaired performance accuracy (Arkell et al., 2020; Hunault et al., 2009; Ramaekers et al., 2011; Ramaekers et al., 2006a; Ramaekers et al., 2006b; Spronk et al., 2016a; Spronk et al., 2016b). Second, THC-containing cannabis led to a significant decline in attention on all 11 tasks that measured any form of attention (Arkell et al., 2020; Englund et al., 2023; Hunault et al., 2009; Mason et al., 2019; 2021; Oliver et al., 2023; Ramaekers et al., 2009; 2011; 2022; Spronk et al., 2016b). 3 tasks (2 sustained attention and 1 divided attention) showed that impaired attention was present after administration of THC-dominant cannabis (Bedrobinol^®^) to occasional but not to chronic cannabis users (compared to placebo), which suggests tolerance to the acute cannabis effects on attention in more long-term cannabis users (Mason et al., 2021; Ramaekers et al., 2009; Ramaekers et al., 2022). Only Ramaekers et al. (2009) included other cognitive measures and demonstrated that occasional users also performed worse on perceptual motor control (critical tracking task) in comparison to placebo. Third, 11 tasks examined the acute effects of THC-containing cannabis on performance of learning and/or memory (Arkell et al., 2020; Englund et al., 2023; Freeman et al., 2021; Hunault et al., 2009; Kloft et al., 2020; Lawn et al., 2023; Mokrysz et al., 2016; Spronk et al., 2016b). THC-containing cannabis caused impaired immediate and delayed recall (Englund et al., 2023; Freeman et al., 2021; Lawn et al., 2023; Mokrysz et al., 2016), and an increase in susceptibility to false memory was demonstrated with THC-dominant cannabis (Bedrobinol^®^) (Kloft et al., 2020). THC-containing cannabis also significantly impaired driving performance as indicated by greater lane weaving during driving compared to placebo (Arkell et al., 2020). Fourth, Arkell and colleagues demonstrated that there were no significant differences between placebo and CBD-dominant cannabis (Bedrolite^®^, 13.75 mg CBD) on the performance of processing speed, memory, attention and planning tasks, nor on driving performance (Arkell et al., 2020). Finally, Hunault et al., 2009 administered cannabis with three different doses of THC (29.3, 49.1, and 69.4 mg) and demonstrated dose-dependent effects on both reaction time and performance of cognitive tasks measuring motor control, attention, and memory, with stronger cognitive impairment after a higher dose. Altogether, these findings imply that THC-containing cannabis can induce slower reaction time and impaired attention and memory. These effects are more pronounced with the administration of cannabis with higher THC doses. CBD-dominant cannabis (Bedrolite^®^) does not seem to affect cognitive function.

#### 3.1.4 Influence of CBD:THC ratios on acute outcome measures

11 studies examined the impact of CBD content on acute outcome measures by comparing the effects of THC-dominant cannabis to those of CBD/THC-containing cannabis, see Table 4. Chester and colleagues investigated the effects of THC-dominant cannabis and CBD/THC-containing cannabis on plasma THC and CBD levels. This study demonstrated that adding 10, 20 or 30 mg CBD to 10 mg THC did not influence THC plasma levels (Chester et al., 2022). CBD had an effect on subjective ratings, which were significantly higher after THC-dominant compared to THC/CBD-containing cannabis that contained the same level of THC (Arkell et al., 2020; Hutten et al., 2022). With regard to the experience of psychotic-like symptoms, THC-dominant cannabis did not differ from CBD/THC-containing cannabis (Englund et al., 2023; Mokrysz et al., 2021). 5 of the 11 studies compared the effects of THC-dominant cannabis to those of CBD/THC-containing cannabis on a variety of cognitive tasks (Arkell et al., 2020; Englund et al., 2023; Lawn et al., 2016; Oliver et al., 2023; Skumlien et al., 2023). On tasks measuring driving performance (Arkell et al., 2020), working memory (spatial N-back and digit span) (Englund et al., 2023), attentional bias (Oliver et al., 2023), verbal learning and verbal episodic memory (Englund et al., 2023; Lawn et al., 2023), no significant differences were found between THC-dominant and CBD/THC-containing cannabis. Lawn and colleagues demonstrated that THC-dominant cannabis induced an overall reduction in motivation as evidenced by a lower likelihood of making a high-effort choice to earn monetary reward. Cannabis with THC and CBD did not appear to reduce this effect but did moderate THC’s effects on expected value to some extent (Lawn et al., 2016). Lastly, neuroimaging studies demonstrated that compared to THC/CBD-equivalent cannabis, administration of THC-dominant cannabis induced a significant reduction in functional connectivity of both the salience network (Wall et al., 2019) and the limbic striatum network, (Wall et al., 2019; Wall et al., 2022), whereas the brain’s anticipatory reward response to money was unaffected (Skumlien et al., 2023). Altogether, when acute effects are compared between THC-dominant and THC/CBD-containing cannabis, it is shown that CBD content may mitigate feelings of anxiety and some of the negative effects of THC-dominant cannabis on functional network connectivity. However, CBD content did not attenuate any of the other acute effects of THC-dominant cannabis, including pharmacokinetics, behavior (psychotic symptoms), subjective experiences (feeling high), and cognition (learning; memory), even up to CBD:THC ratios of 3:1.

### 3.2 The effects of medicinal cannabis in patients

A total of 18 studies investigated the effects of the medicinal use of cannabis in patients with various medical conditions. 11 of these studies assessed subjective pain as the main outcome measure in pain-related disorders such as fibromyalgia, chronic (neuropathic) pain, and chronic migraine. Seven studies focused on other medical conditions including cancer, epileptic etiologies, posttraumatic stress disorder (PTSD), multiple sclerosis, and Alzheimer’s Disease (AD). Bedrocan cannabis variety, dose and route of administration varied greatly between the different studies, see Table 5.

**Table 5:** Overview of studies investigating the effects of medicinal cannabis in various medical conditions.

First author	Study design	Indication	N	Mean age (SD)	M/F	Medication^b^	Route	Frequency	Main effects (group/condition significant effect)	Side effects^c^
**van de Donk et al. (2019)**	DB, PC, Ra, Cr	Fibromyalgia	20	39 (13)	0/20	**Bedrocan** (13.4 mg THC) **Bediol** (13.4 mg THC +17.8 mg CBD) **Bedrolite** (18.4 mg CBD)	Vapor inhalation	Single dose	↑ Pressure pain threshold (Bedrocan and Bediol) = Electrical pain = Spontaneous pain compared	65%–70% coughing during inhalation 25%–35% sore throat and bad taste during inhalation 40%–80% drug high 15%–20% dizziness 5%–30% nausea
**Almog et al. (2020)**	DB, PC, Ra, Cr	Chronic pain	27	48.3 (11.9)	19/8	**Bedrocan** (0.05 mg THC) **Bedrocan** (1.00 mg THC)	Vapor inhalation	Single dose	↓ Pain intensity	20% high 10% cough 9% weakness 8% restlessness 7% dry mouth; dizziness 6% sleepiness 5% nausea 4% moderate decrease in blood pressure
**Aviram et al. (2022)**	RE	Different medical conditions (72% chronic neuropathic pain)	143	62 (17)	77/66	**Bedrocan** (0.25 or 0.5 mg THC) Average daily THC dose: 1.5 ± 0.688 mg THC	Vapor inhalation	Dose and the number of inhalations was individualized	76% of patients reported reduction in pain intensity 92% of patients reported increase quality of life	18% dizziness 11% headache 8% sleepiness
**Baraldi et al. (2022)^a^**	RE	Chronic migraine	32	51.91 (6.51)	5/27	**Bedrocan** (titrated at 19%–22% of THC and <1% of CBD) **Bediol** (titrated at 6.5% of THC and 8% of CBD)	Oral	Daily, for up to 6 months (10–25 drops; 1 mL)	= Number of migraine days per month ↓ Pain intensity ↓ Acute medication taken per month ↓ nausea/vomiting during attacks	31% drowziness 6% postural instability 3% vertigo; weight gain
**Eisenberg et al. (2014)**	OL	Neuropathic pain	8	42 (14)	5/3	**Bedrocan** (3.08 ± 0.02 mg THC)	Vapor inhalation	Single dose	45% reduction in pain intensity	88% lightheadedness for the first 10 min following inhalation
**Giorgi, (2020)**	OB	Fibromyalgia	66	51.9 (11.3)	6/60	**Bedrocan** (night: 10–30 drops) **Bediol** (morning: 10–30 drops) 1 g of cannabis in 10 g of olive oil	Oral	Multiple dose	44% of patients reported improvement of sleep 33% of patients reported improvement of Fibromyalgia	21% dizziness 16% sleepiness 12% palpitations 9% nausea; xerostomia
**Mazza, (2021)^a^**	RE, OL	Fibromyalgia	38	56.6 (9.8)	2/36	**Bedrocan or Bediol** tea: boiling the contents of a sachet (50 or 100 mg) in 200 mL water and 30 mL of milk for 15–20 min oil: 1 g of cannabis in 10 g of olive oil	Oral (tea) Vapor inhalation Sublingual	Multiple dose (2 a day for up to 12 months)	↓ pain intensity at 1, 3, and 12 months 14% decrease in pain intensity of ≥30% 34% decrease in pain intensity of ≥50%	37% mental confusion 14% nausea/vomiting; vertigo/dizziness; restlessness/irritations 11% papilations; somnolence 5% dry mouth; insomnia
**Nunnari et al. (2022)^a^**	RE, OB	Pain-related disorders (37.5% fibromyalgia)	56	44–67 (median 57)	57/15	**Bedrocan** **Bediol**	Oral	Multiple dose (for at least 6 months)	↓ opioid use	NR
**Palmieri et al. (2019)**	RE, OB, OL	Various chronic conditions	20	40.7	8/12	**Bedrocan** (15–30 drops) 5 g Bedrocan in 50 mL of olive oil	Sublingual	Multiple dose (2 times a day for 3 months)	after 6 months ↓ pain ↑ physical ↑ vitality ↑ social functioning ↑ general health state = emotional state monthly reports of psychoactive effects ↑ sleep quality ↑ mood	15% somnolence
**Poli et al. (2018)**	PR	Chronic pain	338	60.9 (14)	115/223	**Bedrocan** (start 5 mg/day THC; after ± 6 months 10 mg/day THC) in 200 mL of boiled water; add 30 mL of milk and simmer for 20 min	Oral	Daily, for up to 1 year	after 12 months ↓ pain intensity ↓ pain disability ↓anxiety and depression symptoms	30% sleepiness 25% mental confusion
**Vulfsons et al. (2020)**	OL	Neuropatic pain	21	44.3 (12.5)	10/11	**Bedrocan** (Median THC per day: 1.5 mg (1–2 mg))	Vapor inhalation	Multiple dose Dose and the number of inhalations was individualized	↓ pain	14% cough immediately following inhalation
**Saccà et al. (2016)**	RE, OB	Multiple sclerosis	13	45.2 (8.02)	8/5	**Bedrocan**	Oral (crumbled on a cookie) Smoking	Daily, for at least 28 days	↓ spasticity	8% dizziness
**Engels et al. (2007)**	OL	Cancer	12 12 **24**	58 55	7/5 7/5	Irinotecan 600 mg i.v. infusion, 3 weeks later with **Bedrocan** 18% THC and 0.8% CBD as 200 mL herbal tea (1000 mg/L) Docetaxel 180 mg i.v. infusion, 3 weeks later with **Bedrocan** 18% THC and 0.8% CBD as 200 mL herbal tea (1000 mg/L)	Oral	Multiple dose (on 15 consecutive days)	Coadministration of medicinal cannabis has no effect on plasma pharmacokinetics	NR
**Zafar et al. (2020)**	RE	A range of epileptic etiologies	10	2–48	NR	**Bedrolite** (CBD ranged from 200mg–550 mg) **Bedica** (THC ranged from 6.6mg–26.5 mg)	Sublingual	Daily	97% mean reduction in monthly seizure frequency	No adverse side effect were reported by carers of the patients
**Zafar et al. (2021)^a^**	RE	A range of epileptic etiologies	10	6.2 (1–13)	NR	**Bedrolite** **Bedica** **Bedrocan**	Sublingual	Daily Mean THC: 5.15 (±6.8) mg Mean CBD: 171.8 (±153.3) mg	86% reduction in seizure frequency	Tiredness
**Pennypacker and Romeo-Sandoval. (2020)**	RE	Drug-resistant epilepsy	5	4–25	0/5	**Bedrocan** (0.6 mg/drop THC; 0.03 mg/drop CBD) **Bedrolite** (0.03 mg/drop THC; 0.24 mg/drop CBD)	Sublingual	Daily (20–30 drops)	60–95 reduction in seizure frequency	One episode of panic attack
**Palmieri and Vadalà. (2023)**	OB	Alzheimer disease	30	69 (36)	9/21	**Bedrocan** 1 g of cannabis in 10 g of olive oil	Sublingual	Twice a day 5 drops and titrates upwards to maximum of 1 mL/day (30 drops) followed by a reduction to 0.5 mL/day at month 3	↓ Agitation ↓ Apathy ↓ Irritability ↓ Sleep disturbances ↓ Eating disturbances ↓ Physically and verbally aggressive behaviours 45% of patients, decrease in cognitive impairment	No side effects occurred
**De Wit et al. (2023)**	QU	Posttraumatic Stress Disorder	18	52 (8.4)	17/1	**Bediol** **Bedrocan** **Bedrolite** **Bedica**	Sublingual Smoking Vapor inhalation	Individualized	↓ Sleep problems ↓ Nightmares ↓ Tension and hyperarousal ↓ Anger and irritability	22% stoned

^a^ Combination treatment with medicinal cannabis from different companies, only Bedrocan products and dosing are reported.

^b^ In bold: cannabis variety; regular: mg THC/CBD.

^c^ Side effects were presented as percentages to simplify comparison between studies. NR, not reported; DB, double blind; PC, placebo controlled; Ra, Randomized; Cr, Crossover; OL, Open-Label; RE, retrospective; PR, prospective; QU, qualitative.

#### 3.2.1 Pain-related medical conditions

Five studies investigated the effects of inhalation of standardized medicinal cannabis (flower material) using a vaporizing device. In four of these studies, using the Syqe inhaler with Bedrocan^®^, patients were asked to rank their pain intensity on a 0 (no pain at all) to 10 (worst possible pain) visual analogue scale (Almog et al., 2020; Aviram et al., 2022; Eisenberg et al., 2014; Vulfsons et al., 2020). All four studies demonstrated a reduction in subjective pain intensity shortly after inhalation, which returned to baseline levels around 90 min post-inhalation (Eisenberg et al., 2014; Vulfsons et al., 2020). In addition, Almog and colleagues (2020) did not report cognitive impairment after inhalation of 0.5 mg (peak THC plasma concentration 14.3 ng/mL) or 1 mg THC (peak THC plasma concentration 33.8 ng/mL), as measured with a cognitive test battery assessing processing speed, memory, and attention. In an experimental study on the effects of inhaled medicinal cannabis in chronic pain patients with fibromyalgia, Van De Donk et al. (2019) demonstrated that compared to placebo, Bedrocan^®^ (22.4 mg THC, <1 mg CBD) and Bediol^®^ (13.4 mg THC, 17.8 mg CBD) caused a significant increase in pressure pain threshold on a pressure pain test that is considered to be a measure of chronic pain. This effect was not seen with Bedrolite^®^ (18.4 mg CBD, <1 mg THC). None of the administered cannabis varieties were effective in reducing spontaneous pain scores more than placebo (Van De Donk et al., 2019). Five studies investigated the impact of cannabis oil administered orally or sublingually processed from Bedrocan^®^ and Bediol^®^ in the context of pain (Baraldi et al., 2022; Giorgi, 2020; Nunnari et al., 2022; Palmieri et al., 2019; Mazza, 2021). Four of these studies reported a decrease in subjective pain intensity (Baraldi et al., 2022; Giorgi, 2020; Palmieri et al., 2019; Mazza, 2021). Baraldi and colleagues (2022) retrospectively studied the effects of 6 months of daily orally administered cannabis oil (Bedrocan^®^ or Bediol^®^; 10–25 drops) in the treatment of chronic migraine. Although the treatment with cannabis oil did not reduce the number of migraines per month, pain intensity, the amount of migraine medication taken and symptoms of nausea and vomiting during attacks were decreased with cannabis treatment. The study by Giorgi, 2020 was an observational study on the effects of 6 months of orally administered cannabis oil (Bedrocan^®^ or Bediol^®^; 10–30 drops) in patients with fibromyalgia. 33% of the patients experienced a reduction in fibromyalgia symptoms after cannabis oil treatment. The retrospective study by Nunnari et al. (2022) investigated the long-term effects of the use of orally administered cannabis oil (Bedrocan^®^ or Bediol^®^) in patients with pain-related disorders that already used cannabis oil for at least 6 months. Although the study did not include pain intensity as an outcome measure, it demonstrated a 23.3% decrease in opioid use during medicinal cannabis treatment. Lastly, one study involved various routes of medicinal cannabis administration. Mazza, 2021 retrospectively studied fibromyalgia patients who received either vaporized whole flower cannabis, cannabis oil extract (sublingually), or cannabis tea, from Bedrocan^®^ and Bediol^®^ for 12 months. Despite an overall drop-out rate of 49% (see paragraph 3.2.3. Side effects), 34% of patients continued medicinal cannabis treatment for 12 months. Of this group, 70% reported a decreased pain intensity of at least 50% without the occurrence of a tolerance effect. Altogether, these studies suggest that treatment with Bedrocan^®^ and/or Bediol^®^ may significantly reduce pain intensity across a broad range of chronic pain-related medical conditions.

#### 3.2.2 Other medical conditions

Three retrospective studies investigated the effect of treatment with medicinal cannabis oils processed from different Bedrocan varieties in severe, intractable epilepsy, mainly in children and adolescents (Pane and Saccà, 2020; Zafar et al., 2020; Zafar et al., 2021). These studies reported a 60%–95% (Pane and Saccà, 2020), 97% (Zafar et al., 2020) and 86% reduction in seizure frequency without the experience of side effects (Pane and Saccà, 2020; Zafar et al., 2020; Zafar et al., 2021). Caregivers of the patients also reported a reduction in panic attacks and insomnia, an increase in cognitive ability and function, and improved behavior (Zafar et al., 2020). Use of anti-epileptic drugs was significantly reduced following initiation of medicinal cannabis treatment. All patients used Bedrolite^®^, but in some patients this was combined with a low dose of Bedica^®^ or Bedrocan^®^ to achieve successful treatment response. Palmieri and Vadalà (2023) investigated the efficacy and safety of 12 weeks of sublingual cannabis oil treatment (derived from Bedrocan^®^) in 20 patients with AD. After 12 weeks of treatment, patients showed significant reductions in symptoms of agitation, apathy, irritability, sleep disturbances, and eating disturbances. Saccà et al. (2016) investigated the impact of treatment with either non-activated oral Bedrocan^®^ cannabis (non-heated, crumbled on a cookie) or smoked Bedrocan^®^ cannabis in a retrospective observational study for at least 28 days in patients with multiple sclerosis that were not responding to treatment with Sativex^®^. Based on a significant reduction in subjective impact of spasticity, 85% (11 patients) were defined as responders to Bedrocan^®^ cannabis after 28 days of therapy, and 70% (9 patients) at follow-up (205 ± 182 days). Lastly, Vermetten and De Wit (2023) conducted semi-structured interviews with veterans with chronic-PTSD that were prescribed off-label medicinal cannabis for symptom relief. Although route of administration and dosages differed between participants, 15 of the 18 patients used (a product derived from) Bediol^®^ cannabis, primarily before bedtime to aid sleep. They reported significant improvements, including increased peace of mind and reduced irritability. Altogether, these studies suggest possible beneficial effects of medicinal cannabis treatment in epilepsy, multiple sclerosis, AD and PTSD.

#### 3.2.3 Side effects

16 studies reported side effects associated with medicinal cannabis treatment, see Table 5 (last column) and Supplementary Material (Supplementary Table S1) for a detailed overview of side effects and drop-out rates. None of the studies reported serious adverse events as response to medicinal cannabis and all side effects were experienced as mild. The most reported side effects included lightheadedness following inhalation (88%; Eisenberg et al., 2014), drug high (20%–80%; Van De Donk et al., 2019; Almog et al., 2020; Vermetten and De Wit, 2023), coughing (10%–70%; Almog et al., 2020; Van De Donk et al., 2019; Vulfsons et al., 2020), mental confusion (25%–37%; Mazza, 2021; Poli et al., 2018), and a sore throat and bad taste during inhalation (25–35%; Van De Donk et al., 2019). Some of these side effects were particularly related to cannabis inhalation, such as coughing (Almog et al., 2020; Van De Donk et al., 2019; Vulfsons et al., 2020), lightheadedness (Almog et al., 2020; Aviram et al., 2022; Eisenberg et al., 2014), or a sore throat (Van De Donk et al., 2019). However, these side effects resolved within minutes after completion of the inhalation procedure. Since only two studies were placebo-controlled (Almog et al., 2020; Van De Donk et al., 2019), it is challenging to distinguish between side effects related to cannabis treatment from those associated with the medical condition itself. There was a large difference in drop-out rates between studies, which appeared to be related to frequency and severity of experienced side effects, see Supplementary Material (Supplementary Table S1). For example, whereas in some studies none of the patients treated with medicinal cannabis exited the study prematurely (Almog et al., 2020; Eisenberg et al., 2014; Palmieri et al., 2019; Palmieri and Vadalà, 2023; Pane and Saccà, 2020; Vulfsons et al., 2020; Zafar et al., 2020; Zafar et al., 2021), Mazza and colleagues (2021) reported that 17 of the initial 35 patients (49%) discontinued cannabis treatment after 3 months, primarily due to mental confusion as a side effect. Studies including patients with more experience with medicinal cannabis use tended to report lower drop-out rates due to side effects (e.g., 1% in Aviram et al., 2022 and 11% discontinued treatment before 6 months in Nunnari et al., 2022). In addition, higher drop-out rates seemed to be related to higher doses of THC. The 49% drop-out rate in the study by Mazza and colleagues (2021) coincided with an average 55 mg THC/day, whereas Aviram et al. (2022) reported a 1% drop-out rate and an average 1.5 mg THC/day. It can therefore be speculated that high dosages may result in more side effects and a higher drop-out rate. Altogether, medicinal cannabis appeared to have a mild safety profile. During vapor inhalation, coughing was among the most frequently reported side effects. In addition, lightheadedness, drug high, and mental confusion were frequently reported regardless of route of administration. Two possible factors involved in the experience of side effects and thus in drop-out rates were cannabis use history as well as a higher dose of THC.

### 3.3 Case studies

Five papers reported individual case studies that involved medicinal cannabis treatment (Table 6). Two case studies were related to the treatment of Tourette’s syndrome (Jakubovski and Müller-Vahl, 2017; Szejko et al., 2019). Both patients received the Bedrocan^®^ cannabis variety through vapor inhalation, which resulted in a 70% reduction of tics in one patient after 8 months (100 mg, increased to 600 mg daily; Jakubovski and Müller-Vahl, 2017) and a complete remission of tics for the other after 1.5 months (20 mg daily; Szejko et al., 2019). Only the former patient experienced a feeling of high after administration in the first few weeks which completely disappeared later in treatment, which may have been related to the higher cannabis starting dose. Lastly, three case studies reported reduced symptoms and improved functioning associated with medical cannabis inhalation with Bedrocan^®^, Bediol^®^ or Bedica^®^ in patients with treatment-resistant stuttering, attention deficit hyperactivity disorder and Hypermobile Ehlers-Danlos syndrome, respectively (Dar, 2021; Hupli, 2019; Szejko et al., 2021). Altogether, these case studies provide indications for the use of medicinal cannabis across a broad spectrum of medical conditions. Future research should however focus on conducting clinical randomized trials to systematically determine whether patients suffering from these medical conditions could benefit from treatment with standardized medicinal cannabis.

**TABLE 6:** | Overview of case study investigating the effects medicinal cannabis in various medical conditions.

First Author	Age	M/F	Indication	Medication**	Route	Frequency	Main effect(s)	Side effects
Jakubovski et al. (2017)	19	M	Tourette syndrome	**Bedrocan (100-600 mg)**	Vapor inhalation	Daily	Able to speak nearly fluently in most situations. Improvement also occurred in other tics, such as head nodding. A significant tic reduction of about 70% including the blocking speech tics and a feeling of “being calmer” throughout the whole day.	In the first few weeks: a “high” after administration (disappeared later in treatment).
Dar (2021)*	18	F	Hypermobile Ehlers- Danlos syndrome	**Bedrocan and Bedica**	Vapor inhalation	NR	Improvement in quality of life. Able to start physiotherapy and regain muscle strength, whereas previously, unable to complete exercises. Better pain management, facilitating increased participation in psychotherapy. The frequency and extent of joint dislocation declined. Was able to, at first, manoeuvre wheelchair with greater ease for longer distances and, in time, relearn how to walk.	During the titration period: trouble concentrating, nausea, sedation and highs.
Hupli et al. (2018)	33	M	Attention deficit hyperactivity disorder	**Bedrocan and Bediol (1000-2000 mg)** 2:1 ratio	Vapor inhalation	Bedrocan in the morning Bediol in the evening	Bedrocan: a positive impact on ADHD symptoms, reducing hyperactivity, improving focus and impulse control, and giving better tolerance to frustration. After adverse reactions Bediol was added. Bediol: anxiety reducing, and sleeping pattern improved significantly; fall asleep quickly and sleep through the night.	During a period of increased stress the use of Bedrocan began to induce sleeping problems and agitation.
Szejko et al. (2019)*	12	M	Tourette syndrome	**Bedrocan 20 mg** 4.4 mg THC	Vapor inhalation	Twice a day	Immediate and nearly complete remission of tics, fall asleep without problems tics, premonitory urges, and overall impairment significantly improved.	No side effects occurred
Szejko et al. (2021)	20	M	Treatment-resistant stuttering	**Bedrocan (300–700 mg/day)** **Bediol (50 mg/day)**	Vapor inhalation	NR	Improved speech fluency, remission of (social) anxiety, improved mood, and reduced stress, resulting in an overall improvement of quality of life. In addition, improved attention, concentration, and sleep, increased self-confidence, and better social life.	No side effects occurred

*Combination treatment with medicinal cannabis from different companies, only Bedrocan products and dosing are reported; **In bold: cannabis variety; regular: mg THC/CBD.

NR, Not Reported.

## 4 Discussion

The current systematic literature review is the first to provide an overview of the effects of standardized cannabis products in healthy volunteers and patients. Studies were included that investigated the impact of cannabis products (e.g., herbal cannabis, oils) derived from cannabis varieties cultivated by Bedrocan, a leading Dutch producer of pharmaceutical-quality herbal standardized medicinal cannabis. Our findings suggest that cannabis administration to healthy volunteers induces dose-dependent acute effects, such as rapidly rising THC and CBD blood concentrations, the subjective experience of high and anxiety, and a slower reaction time and impaired accuracy of divided and sustained attention, learning and working memory tasks. Patient studies suggest that treatment with medicinal cannabis reduces pain intensity across a broad range of chronic pain-related medical conditions. In general, medicinal cannabis appears to have a mild safety profile, with some minor side effects such as feeling high, coughing and mental confusion. These side effects were often more intense with high doses of THC and certain side effects (e.g., coughing, lightheadedness and a sore throat) were particularly related to inhalation as route of administration.

Findings of dose-dependent acute kinetic, subjective and cognitive effects of standardized cannabis in healthy volunteers are consistent with those reported in other recent reviews (Freeman et al., 2019b; Kroon et al., 2021; Xiao et al., 2023; Zamarripa et al., 2022; Zhornitsky et al., 2021). For example, in line with our findings, Zamarripa et al. (2022) reported peak THC plasma levels within 30 min after vaporizing or smoking cannabis, which returned to baseline after approximately 4 h. This was accompanied by the subjective experience of feeling high at or shortly after reaching peak plasma levels. In addition, both the reviews of Kroon et al. (2021) and Zhornitsky et al. (2021) further underlined that the strongest effects of cannabis administration on cognition are found on the domains of attention, learning and memory. These findings imply that possible adverse effects associated with medicinal cannabis treatment may include feelings of high and anxiety as well as longer reaction times and an attenuated cognitive ability. Because of the demonstrated dose-dependent decline of cognition and increase in feeling high, this may be particularly relevant for cannabis with higher doses of THC (Hunault et al., 2008; 2009; 2014).

Studies investigating the role of CBD:THC ratios in the acute effects of cannabis demonstrated that although CBD may mitigate feelings of anxiety and some of the negative effects of THC on functional network connectivity, CBD did not modulate any of the other acute effects of THC, including behavior, subjective experiences and cognition, even up to CBD:THC ratios of 3:1. A recent systematic review from Freeman and colleagues (2019a) converged on a similar conclusion, that co-administration with CBD may attenuate THC-induced feelings of anxiety and psychotic-like effects, but not subjective intoxicating, psychomotor or cognitive effects. Their review focused on the influence of CBD on THC effects and concluded that CBD primarily reduced the acute effects of THC. Although effects were mixed, fewer participants experienced extreme feelings of anxiety and psychotic-like effects when THC was co-administered with CBD. However, there were no differences in subjective intoxicating effects, psychomotor effects or cognitive effects. In addition, previous reviews by Pennypacker and Romero-Sandoval (2020) and Iseger and Bossong (2015) suggest that the modulating effect of CBD may depend on factors such as CBD:THC ratio, time of administration (concomitant or at separated times) and patients’ history of cannabis use. For example, Dalton and colleagues (1976) showed that simultaneous inhalation of CBD (150 μg/kg) and THC (25 μg/kg; CBD:THC ratio 6:1) attenuated the subjective euphoria of THC and caused a trend towards a decrease in THC-induced psychomotor impairment. However, in the same study, pretreatment with CBD did not alter the effects of THC (Dalton et al., 1976). In addition, a study by Solowij et al. (2019) demonstrated that low doses of vaporized CBD (4 mg) enhanced the intoxicating effects of THC (8 mg; CBD:THC ratio 2:1), whereas high doses of vaporized CBD (400 mg) reduced the intoxicating effects of THC (8 mg; CBD:THC ratio 50:1). Their findings provide evidence that the possible attenuation of THC effects is dependent upon the ratio of CBD:THC. In the current review, most studies used a 1:1 CBD:THC ratio, with a maximum of 3:1, which may account for the reported null effects. Therefore, future studies are warranted to disentangle the complex relationship between CBD and THC and investigate how CBD can impact potential adverse effects of THC.

Our findings indicate that treatment with Bedrocan^®^ and/or Bediol^®^ reduces pain intensity across a broad range of chronic pain-related medical conditions, which is generally accompanied by mild and transient adverse effects such as coughing, lightheadedness, feeling high and mental confusion. Although systematic reviews and meta-analyses that included randomized clinical trials on the efficacy of medicinal cannabis for pain-related conditions demonstrated mixed results, it appeared that successful treatment may depend on the particular pain condition, route of cannabis administration and cannabis composition (Fisher et al., 2021; Jeddi et al., 2024; Longo et al., 2021; Sainsbury et al., 2021). In particular, stronger and more consistent effects of cannabis treatment were shown in patients with neuropathic pain, for inhalation as route of administration and with THC-containing products (unlike higher-CBD cannabis products) (Longo et al., 2021; Sainsbury et al., 2021). In our review however both inhalation and oral administration routes demonstrated positive effects on subjective pain intensity in a variety of pain-related medical conditions. This decrease in pain intensity was demonstrated for the Bedrocan^®^ (THC 22%; CBD <1.0%) and Bediol^®^ (THC 6.3%; CBD 8%) cannabis varieties or a combination of both. The only study that investigated the effects of various cannabis varieties on experimental measures of pain demonstrated that both Bedrocan^®^ and Bediol^®^ but not Bedrolite^®^ (THC <1.0%; CBD 7.5%) increased the pressure pain threshold (Van De Donk et al., 2019). These results are consistent with a recent survey on patient experiences with the use of medicinal cannabis in the Netherlands demonstrating that 60% of patients used medicinal cannabis for chronic pain, primarily Bedrocan^®^ cannabis flos and/or Bediol^®^ derived cannabis oil (Ekhart et al., 2023). However, because the vast majority of available data in our review was obtained retrospectively, randomized clinical trials are needed to draw further conclusions about safety and efficacy of medicinal cannabis in the treatment of chronic pain.

Studies discussed in the current systematic review show important differences in methodology, which may hamper the interpretation of the results. First, the route of administration varies between healthy volunteers (all studies smoking or vapor inhalation) and patients (50% of studies sublingually/orally). This makes it difficult to translate findings from healthy volunteers to clinical populations, because effects after smoking or inhalation occur rapidly and are short-lived, while the effects of oral forms are delayed and more prolonged due to slower absorption and lower peak levels of THC/CBD (Grotenhermen, 2003). It is important to take this into consideration for different medical conditions to reach the desired effect. Smoking cannabis is highly discouraged in patients given the harmful effects especially when mixed with tobacco (Chaiton et al., 2022). In addition, the actual composition of products (e.g., oil) that use Bedrocan flower as the base for cannabis products is not always reported. Therefore, results of these studies should be interpreted with caution. Future studies should be clear and transparent about CBD and THC content of cannabis medication that is prescribed. Second, study participants differed in their history of cannabis use, which impacted on the assessment of acute cannabis effects. For example, it was demonstrated that chronic cannabis users displayed higher THC blood concentrations, experienced a lesser degree of feeling high, and less pronounced cognitive deficits on attention and motor inhibition tasks than occasional cannabis users (Fabritius et al., 2013; Mason et al., 2021; Ramaekers et al., 2009; Ramaekers et al., 2022). In addition, all healthy participants that were included had experience with the use of cannabis which may result in a bias toward individuals who have more positive experiences with cannabis. In patients, cannabis use history may be related to the occurrence of side effects and drop-out rates, with less experienced patients being more likely to report more adverse cannabis effects. Third, higher drop-out rates may be related to higher doses of THC. The 49% drop-out rate in the study by Mazza and colleagues (2021) coincided with an average 55 mg THC/day, whereas Aviram et al. (2022) reported a 1% drop-out rate and an average 1.5 mg THC/day. More research is needed to identify the impact of route of administration, cannabis use history and cannabis dose on side effects and drop-out rates. Fourth, possible differences between men and women in the effects of cannabis may have influenced our findings. Remarkably, in the current systematic review, 71% of all healthy participants were male, compared to 40% of participants in the patient studies. Only one study that exclusively included male participants gave a rationale for this choice (Mokrysz et al., 2016). This study compared the acute effects of cannabis between adults and adolescents and recruited only males due to the different ages of puberty onset and the potentially different brain development trajectories between sexes. One additional reason for the lower inclusion rate of women in the cannabis studies discussed in the current review could be found in the potential impact of female hormones and the menstrual cycle on outcome measures (Fattore and Fratta, 2010), although this is no longer considered a justifiable reason for exclusion and thus not currently acceptable practice. Various studies with the administration of non-Bedrocan cannabis products demonstrated sex differences in the acute effects of cannabis (Remaekers et al., 2006a; Haney, 2007; Sholler et al., 2021). For example, Sholler et al. (2021) showed that female participants exhibited greater sensitivity to subjective cannabis effects. The only study included in the current review that addressed sex differences in acute cannabis effects did not demonstrate systematic differences between males and females (Arkell et al., 2022). Therefore, more research is warranted to further investigate sex differences in the safety and efficacy of medicinal cannabis, particularly since the most frequent chronic pain conditions occur more often in females than males (Fillingim, 2023). Fifth, age difference is also an important factor to consider in future research. As our review already demonstrated, the subjective measures of stoned, drug effect and anxiety were stronger in adults than in adolescents following Bedrobinol^®^ cannabis administration (Mokrysz et al., 2016; Mokrysz et al., 2021). Also cognitive differences were found with Bedrobinol^®^ cannabis, with adults demonstrating a slower reaction time on a memory task in comparison to adolescents, but adolescents demonstrated less accuracy in response inhibition on a stop signaling tasks than adults (Mokrysz et al., 2016). In addition, it is also crucial to focus on the effects in older adults because they show the largest increase in cannabis use (Mueller et al., 2021).

Lastly, although we only selected standardized cannabis from Bedrocan for this review, there was still a large difference in the variety, dosage and route of administration used among the included studies. Therefore, comparing the diverse study results proved challenging, especially given the differing methodologies used across the studies. Finding more uniformity in variety, dose, and outcome measures will make it easier to compare future research. This is especially important since conducting research with a uniform design on cannabis with a consistently stable composition will provide reliable data on the safety and efficacy of specific cannabis cultivars in treating different medical conditions.

In conclusion, studies investigating the impact of standardized Bedrocan cannabis products demonstrated dose-dependent acute effects in healthy volunteers, including subjective experiences and diminished cognitive function. Medicinal cannabis treatment reduced pain intensity across a broad range of chronic pain-related medical conditions, with only mild and transient side effects. Safety and efficacy of medicinal cannabis should be further investigated in randomized clinical trials with sufficient sample size, with particular focus on cannabis dose and composition, age and differences between males and females.

## Data Availability

The original contributions presented in the study are included in the article/Supplementary Material, further inquiries can be directed to the corresponding author.

## References

[B1] Almog S. Aharon-Peretz J. Vulfsons S. Ogintz M. Abalia H. Lupo T. (2020). The pharmacokinetics, efficacy, and safety of a novel selective-dose cannabis inhaler in patients with chronic pain: a randomized, double-blinded, placebo-controlled trial. Eur. J. Pain (United Kingdom) 24 (8), 1505–1516. 10.1002/ejp.1605 PMC7496774 32445190

[B2] Arkell T. R. Kevin R. C. Vinckenbosch F. Lintzeris N. Theunissen E. Ramaekers J. G. (2022). Sex differences in acute cannabis effects revisited: results from two randomized, controlled trials. Addict. Biol. 27 (2), e13125. 10.1111/adb.13125 34936167 PMC9286641

[B3] Arkell T. R. Vinckenbosch F. Kevin R. C. Theunissen E. L. McGregor I. S. Ramaekers J. G. (2020). Effect of cannabidiol and ?9-Tetrahydrocannabinol on driving performance: a randomized clinical trial. JAMA - J. Am. Med. Assoc. 324 (21), 2177–2186. 10.1001/jama.2020.21218 PMC7709000 33258890

[B4] Arzimanoglou A. Brandl U. Cross J. H. Gil-Nagel A. Lagae L. Landmark C. J. (2020). Epilepsy and cannabidiol: a guide to treatment. Epileptic Disord. 22 (1), 1–14. 10.1684/EPD.2020.1141 32096470

[B6] Aviram J. Atzmony D. Eisenberg E. (2022). Long-term effectiveness and safety of medical cannabis administered through the metered-dose Syqe Inhaler. Pain Rep. 7 (3), E1011. 10.1097/PR9.0000000000001011 35620248 PMC9116950

[B7] Baraldi C. Lo Castro F. Negro A. Ferrari A. Cainazzo M. M. Pani L. (2022). Oral cannabinoid preparations for the treatment of chronic migraine: a retrospective study. Pain Med. 23 (2), 396–402. 10.1093/pm/pnab245 34347088

[B8] Baratta F. Pignata I. Ravetto Enri L. Brusa P. (2022). Cannabis for medical use: analysis of recent clinical trials in view of current legislation. Front. Pharmacol. 13, 888903. 10.3389/fphar.2022.888903 35694246 PMC9174563

[B9] Battistella G. Fornari E. Thomas A. Mall J. F. Chtioui H. Appenzeller M. (2013). Weed or wheel! fMRI, behavioural, and toxicological investigations of how cannabis smoking affects skills necessary for driving. PLoS ONE 8 (1), e52545. 10.1371/journal.pone.0052545 23300977 PMC3534702

[B10] Bhaskar A. Bell A. Boivin M. Briques W. Brown M. Clarke H. (2021). Consensus recommendations on dosing and administration of medical cannabis to treat chronic pain: results of a modified Delphi process. J. Cannabis Res. 3 (1), 22. 10.1186/s42238-021-00073-1 34215346 PMC8252988

[B11] Bilbao A. Spanagel R. (2022). Medical cannabinoids: a pharmacology-based systematic review and meta-analysis for all relevant medical indications. BMC Med. 20 (Issue 1), 259. 10.1186/s12916-022-02459-1 35982439 PMC9389720

[B102] Brenneisen R. Meyer P. Chtioui H. Saugy M. Kamber M. (2010). Plasma and urine profiles of Δ 9-tetrahydrocannabinol and its metabolites 11-hydroxy-Δ 9-tetrahydrocannabinol and 11-nor-9-carboxy-Δ 9-tetrahydrocannabinol after cannabis smoking by male volunteers to estimate recent consumption by athletes. Anal. Bioanal. Chem. 396 (7), 2493–2502. 10.1007/s00216-009-3431-3 20112012

[B12] Carter G. T. Weydt P. Kyashna-Tocha M. Abrams D. I. Francisco S. (2004). Medicinal cannabis: rational guidelines for dosing. Muraco. Org. 7, 464–470. Available at: https://www.muraco.org/resources/2004%20Carter%20Weydt%20Kyashna-Tocha%20Abrams%20MJ%20Dosing.pdf. 15154108

[B13] Chaiton M. Kundu A. Rueda S. Di Ciano P. (2022). Are vaporizers a lower-risk alternative to smoking cannabis? Can. J. Public Health 113 (2), 293–296. 10.17269/s41997-021-00565-w 34448130 PMC8975973

[B14] Chester L. A. Englund A. Chesney E. Oliver D. Wilson J. Sovi S. (2022). Effects of cannabidiol and delta-9-tetrahydrocannabinol on plasma endocannabinoid levels in healthy volunteers: a randomized double-blind four-arm crossover study. Cannabis Cannabinoid Res. 9, 188–198. 10.1089/can.2022.0174 36493386 PMC10874814

[B15] Cooper Z. D. Abrams D. I. Gust S. Salicrup A. Throckmorton D. C. (2021). Challenges for clinical cannabis and cannabinoid research in the United States. JNCI Monogr. 2021 (58), 114–122. 10.1093/JNCIMONOGRAPHS/LGAB009 PMC8783595 34850896

[B16] Dalton W. S. Martz R. Lemberger L. Rodda B. E. Forney R. B. (1976). Influence of cannabidiol on delta-9-tetrahydrocannabinol effects. Clin. Pharmacol. and Ther. 19 (3), 300–309. 10.1002/CPT1976193300 770048

[B17] Dar S. (2021). Treating pain related to Ehlers-Danlos syndrome with medical cannabis. BMJ Case Rep. 14 (7), e242568. 10.1136/bcr-2021-242568 PMC8728473 34301703

[B18] de Bruijn S. E. M. de Graaf C. Witkamp R. F. Jager G. (2017). Explorative placebo-controlled double-blind intervention study with low doses of inhaled δ9-tetrahydrocannabinol and cannabidiol reveals No effect on sweet taste intensity perception and liking in humans. Cannabis Cannabinoid Res. 2 (1), 114–122. 10.1089/can.2017.0018 28861511 PMC5569584

[B19] Eisenberg E. Ogintz M. Almog S. (2014). The pharmacokinetics, efficacy, safety, and ease of use of a novel portable metered-dose cannabis inhaler in patients with chronic neuropathic pain: a phase 1a study. J. Pain Palliat. Care Pharmacother. 28 (3), 216–225. 10.3109/15360288.2014.941130 25118789

[B20] Ekhart C. Schipper S. J. V. Velde M. J. van de Rolfes L. Kant A. (2023). Patient experiences with the use of medicinal cannabis in The Netherlands: a cohort-event monitoring study. Available at: Https://Home.Liebertpub.Com/Imr. 2, 138–146. 10.1089/IMR.2023.0036

[B104] Engels F. K. De Jong F. A. Sparreboom A. Mathot R. A. Loos W. J. Kitzen J. J. (2007). Medicinal cannabis does not influence the clinical pharmacokinetics of irinotecan and docetaxel. Oncologist 12 (3), 291–300. 10.1634/theoncologist.12-3-291 17405893

[B21] Englund A. Oliver D. Chesney E. Chester L. Wilson J. Sovi S. (2023). Does cannabidiol make cannabis safer? A randomised, double-blind, cross-over trial of cannabis with four different CBD:THC ratios. Neuropsychopharmacology 48 (6), 869–876. 10.1038/s41386-022-01478-z 36380220 PMC10156730

[B22] Fabritius M. Chtioui H. Battistella G. Annoni J. M. Dao K. Favrat B. (2013). Comparison of cannabinoid concentrations in oral fluid and whole blood between occasional and regular cannabis smokers prior to and after smoking a cannabis joint. Anal. Bioanal. Chem. 405 (30), 9791–9803. 10.1007/s00216-013-7412-1 24202191

[B23] Farrah K. Young K. Tunis M. C. Zhao L. (2019). Risk of bias tools in systematic reviews of health interventions: an analysis of PROSPERO-registered protocols. Syst. Rev. 8 (1), 280–289. 10.1186/s13643-019-1172-8 31730014 PMC6857304

[B24] Fattore L. Fratta W. (2010). How important are sex differences in cannabinoid action. Br. J. Pharmacol. 160 (3), 544–548. 10.1111/j.1476-5381.2010.00776.x 20590564 PMC2931555

[B25] Fillingim R. B. (2023). Sex, gender, and pain. Princ. Gender-Specific Med. Sex Gender-Specific Biol. Postgenomic Era, 769–792. 10.1016/B978-0-323-88534-8.00011-0

[B26] Fisher E. Moore R. A. Fogarty A. E. Finn D. P. Finnerup N. B. Gilron I. (2021). Cannabinoids, cannabis, and cannabis-based medicine for pain management: a systematic review of randomised controlled trials. Pain 162, S45–S66. 10.1097/j.pain.0000000000001929 32804836

[B27] Freeman A. M. Mokrysz C. Hindocha C. Lawn W. Morgan C. J. A. Freeman T. P. (2021). Does variation in trait schizotypy and frequency of cannabis use influence the acute subjective, cognitive and psychotomimetic effects of delta-9-tetrahydrocannabinol? A mega-analysis. J. Psychopharmacol. 35 (7), 804–813. 10.1177/0269881120959601 33427016

[B28] Freeman A. M. Petrilli K. Lees R. Hindocha C. Mokrysz C. Curran H. V. (2019a). How does cannabidiol (CBD) influence the acute effects of delta-9-tetrahydrocannabinol (THC) in humans? A systematic review. Neurosci. and Biobehav. Rev. 107, 696–712. 10.1016/J.NEUBIOREV.2019.09.036 31580839

[B29] Freeman A. M. Petrilli K. Lees R. Hindocha C. Mokrysz C. Curran H. V. (2019b). How does cannabidiol (CBD) influence the acute effects of delta-9-tetrahydrocannabinol (THC) in humans? A systematic review. Neurosci. Biobehav. Rev. 107, 696–712. 10.1016/j.neubiorev.2019.09.036 31580839

[B30] Giorgi, Clinical and experimental rheumatology. (2020).

[B31] Green B. Kavanagh D. J. Young R. M. (2004). Reasons for cannabis use in men with and without psychosis. Drug Alcohol Rev. 23 (4), 445–453. 10.1080/09595230412331324563 15763749

[B32] Grotenhermen F. (2003). Pharmacokinetics and pharmacodynamics of cannabinoids. Clin. Pharmacokinet. 42, 327–360. 10.2165/00003088-200342040-00003 12648025

[B99] Haney M. (2007). Opioid antagonism of cannabinoid effects: differences between marijuana smokers and nonmarijuana smokers. Neuropsychopharmacology 32 (6), 1391–1403. 10.1038/sj.npp.1301243 17091128

[B34] Hunault C. C. Böcker K. B. E. Stellato R. K. Kenemans J. L. De Vries I. Meulenbelt J. (2014). Acute subjective effects after smoking joints containing up to 69 mg Δ9-tetrahydrocannabinol in recreational users: a randomized, crossover clinical trial. Psychopharmacology 231 (24), 4723–4733. 10.1007/s00213-014-3630-2 24879495

[B35] Hunault C. C. Mensinga T. T. Böcker K. B. E. Schipper C. M. A. Kruidenier M. Leenders M. E. C. (2009). Cognitive and psychomotor effects in males after smoking a combination of tobacco and cannabis containing up to 69 mg delta-9-tetrahydrocannabinol (THC). Psychopharmacology 204 (1), 85–94. 10.1007/s00213-008-1440-0 19099294

[B36] Hunault C. C. Mensinga T. T. De Vries I. Kelholt-Dijkman H. H. Hoek J. Kruidenier M. (2008). Delta-9-tetrahydrocannabinol (THC) serum concentrations and pharmacological effects in males after smoking a combination of tobacco and cannabis containing up to 69 mg THC. Psychopharmacology 201 (2), 171–181. 10.1007/s00213-008-1260-2 18695931

[B37] Hupli A. M. M. (2019). Medical cannabis for adult attention deficit hyperactivity disorder: sociological patient case report of cannabinoid therapeutics in Finland. Med. Cannabis Cannabinoids 1 (2), 112–118. 10.1159/000495307 34676327 PMC8489316

[B38] Hutten N. R. P. W. Arkell T. R. Vinckenbosch F. Schepers J. Kevin R. C. Theunissen E. L. (2022). Cannabis containing equivalent concentrations of delta-9-tetrahydrocannabinol (THC) and cannabidiol (CBD) induces less state anxiety than THC-dominant cannabis. Psychopharmacology 239 (11), 3731–3741. 10.1007/s00213-022-06248-9 36227352 PMC9584997

[B40] Iseger T. A. Bossong M. G. (2015). A systematic review of the antipsychotic properties of cannabidiol in humans. Schizophrenia Res. 162 (1–3), 153–161. 10.1016/J.SCHRES.2015.01.033 25667194

[B41] Jakubovski E. Müller-Vahl K. (2017). Speechlessness in Gilles de la Tourette Syndrome: Cannabis-based medicines improve severe vocal blocking tics in two patients. Int. J. Mol. Sci. 18 (8), 1739. 10.3390/ijms18081739 28796166 PMC5578129

[B42] Jeddi H. M. Busse J. W. Sadeghirad B. Levine M. Zoratti M. J. Wang L. (2024). Cannabis for medical use versus opioids for chronic non-cancer pain: a systematic review and network meta-analysis of randomised clinical trials. BMJ Open 14, e068182. 10.1136/bmjopen-2022-068182 PMC10773353 38171632

[B43] Karila L. Roux P. Rolland B. Benyamina A. Reynaud M. Aubin H.-J. (2014). Acute and long-term effects of cannabis use: a review. Curr. Pharm. Des. 20 (25), 4112–4118. 10.2174/13816128113199990620 24001294

[B44] Kloft L. Otgaar H. Blokland A. Monds L. A. Toennes S. W. Loftus E. F. (2020). Cannabis increases susceptibility to false memory. Proc. Natl. Acad. Sci. U. S. A. 117 (9), 4585–4589. 10.1073/pnas.1920162117 32041881 PMC7060677

[B45] Kowal M. A. Hazekamp A. Colzato L. S. Van Steenbergen H. Van Der Wee N. J. A. Durieux J. (2015a). Cannabis and creativity: highly potent cannabis impairs divergent thinking in regular cannabis users. Psychopharmacology 232 (6), 1123–1134. 10.1007/s00213-014-3749-1 25288512 PMC4336648

[B46] Kowal M. A. van Steenbergen H. Colzato L. S. Hazekamp A. van der Wee N. J. A. Manai M. (2015b). Dose-dependent effects of cannabis on the neural correlates of error monitoring in frequent cannabis users. Eur. Neuropsychopharmacol. 25 (11), 1943–1953. 10.1016/j.euroneuro.2015.08.001 26298832

[B47] Kroon E. Kuhns L. Cousijn J. (2021). The short-term and long-term effects of cannabis on cognition: recent advances in the field. Curr. Opin. Psychol. 38, 49–55. 10.1016/j.copsyc.2020.07.005 32823178

[B48] Lawn W. Freeman T. P. Pope R. A. Joye A. Harvey L. Hindocha C. (2016). Acute and chronic effects of cannabinoids on effort-related decision-making and reward learning: an evaluation of the cannabis ‘amotivational’ hypotheses. Psychopharmacology 233 (19–20), 3537–3552. 10.1007/S00213-016-4383-X 27585792 PMC5021728

[B49] Lawn W. Trinci K. Mokrysz C. Borissova A. Ofori S. Petrilli K. (2023). The acute effects of cannabis with and without cannabidiol in adults and adolescents: a randomised, double-blind, placebo-controlled, crossover experiment. Addiction 118 (7), 1282–1294. 10.1111/add.16154 36750134 PMC10481756

[B50] Longo R. Oudshoorn A. Befus D. (2021). Cannabis for chronic pain: a rapid systematic review of randomized control trials. Pain Manag. Nurs. 22 (2), 141–149. 10.1016/j.pmn.2020.11.006 33353819

[B51] Mané A. Fernández-Expósito M. Bergé D. Gómez-Pérez L. Sabaté A. Toll A. (2015). Relationship between cannabis and psychosis: reasons for use and associated clinical variables. Psychiatry Res. 229 (1–2), 70–74. 10.1016/J.PSYCHRES.2015.07.070 26235479

[B52] Mason N. L. Theunissen E. L. Hutten N. R. P. W. Tse D. H. Y. Toennes S. W. Jansen J. F. A. (2021). Reduced responsiveness of the reward system is associated with tolerance to cannabis impairment in chronic users. Addict. Biol. 26 (1), e12870. 10.1111/adb.12870 31865628 PMC7757162

[B53] Mason N. L. Theunissen E. L. Hutten N. R. P. W. Tse D. H. Y. Toennes S. W. Stiers P. (2019). Cannabis induced increase in striatal glutamate associated with loss of functional corticostriatal connectivity. Eur. Neuropsychopharmacol. 29 (2), 247–256. 10.1016/j.euroneuro.2018.12.003 30553697

[B54] Mazza M. (2021). Medical cannabis for the treatment of fibromyalgia syndrome: a retrospective, open-label case series. J. Cannabis Res. 3 (1), 4. 10.1186/s42238-021-00060-6 33597032 PMC7890993

[B55] Mokrysz C. Freeman T. P. Korkki S. Griffiths K. Curran H. V. (2016). Are adolescents more vulnerable to the harmful effects of cannabis than adults? A placebo-controlled study in human males. Transl. Psychiatry 6 (11), e961. 10.1038/tp.2016.225 27898071 PMC5290352

[B56] Mokrysz C. Shaban N. D. C. Freeman T. P. Lawn W. Pope R. A. Hindocha C. (2021). Acute effects of cannabis on speech illusions and psychotic-like symptoms: two studies testing the moderating effects of cannabidiol and adolescence. Psychol. Med. 51 (12), 2134–2142. 10.1017/S0033291720001038 32340632

[B57] Mueller R. L. Ellingson J. M. Cinnamon Bidwell L. Bryan A. D. Hutchison K. E. (2021). Are the acute effects of THC different in aging adults? Brain Sci. 11 (5), 590. 10.3390/BRAINSCI11050590 34062795 PMC8147270

[B58] Niesink R. J. M. van Laar M. W. (2013). Does cannabidiol protect against adverse psychological effects of THC? Front. Psychiatry 4, 130. 10.3389/fpsyt.2013.00130 24137134 PMC3797438

[B59] Nunnari P. Ladiana N. Ceccarelli G. Notaro P. (2022). Long-term Cannabis-based oil therapy and pain medications prescribing patterns: an Italian observational study. Eur. Rev. Med. Pharmacol. Sci. 26 (4), 1224–1234. 10.26355/eurrev_202202_28114 35253178

[B60] Oliver D. Englund A. Chesney E. Chester L. Wilson J. Sovi S. (2023). Cannabidiol does not attenuate acute delta-9-tetrahydrocannabinol-induced attentional bias in healthy volunteers: a randomised, double-blind, cross-over study. Addiction 119, 322–333. 10.1111/add.16353 37821096 PMC10952555

[B62] Page M. J. McKenzie J. E. Bossuyt P. M. Boutron I. Hoffmann T. C. Mulrow C. D. (2021). The PRISMA 2020 statement: an updated guideline for reporting systematic reviews. BMJ 372, n71. 10.1136/BMJ.N71 33782057 PMC8005924

[B63] Palmieri B. Laurino C. Vadalà M. (2019). Spontaneous, anecdotal, retrospective, open-label study on the efficacy, safety and tolerability of cannabis galenical preparation (Bedrock). Int. J. Pharm. Pract. 27 (3), 264–270. 10.1111/ijpp.12514 30768819 PMC6593769

[B64] Palmieri B. Vadalà M. (2023). Oral thc: cbd cannabis extract in main symptoms of Alzheimer disease: agitation and weight loss. Clin. Ter. 174 (1), 53–60. 10.7417/CT.2023.5009 36655645

[B65] Pane C. Saccà F. (2020). The use of medical grade cannabis in Italy for drug-resistant epilepsy: a case series. Neurol. Sci. 41 (3), 695–698. 10.1007/s10072-019-04162-1 31776867

[B66] Pennypacker S. D. Romero-Sandoval E. A. (2020). CBD and THC: do they complement each other like yin and yang? Pharmacotherapy 40 (11), 1152–1165. NLM (Medline). 10.1002/phar.2469 33080058

[B33] Pertwee R. G. (Editor) (2014). Handbook of cannabis. United States: Oxford University Press.

[B67] Poli P. Crestani F. Salvadori C. Valenti I. Sannino C. (2018). Medical cannabis in patients with chronic pain: effect on pain relief, pain disability, and psychological aspects. A prospective non randomized single arm clinical trial. Clin. Ter. 169 (3), E102–E107. 10.7417/T.2018.2062 29938740

[B68] Quality standards: GMP and GMCCP (2024). Available at: https://bedrocan.com/about-us/quality-standards/.

[B69] Quigley J. M. Thompson J. C. Halfpenny N. J. Scott D. A. (2019). Critical appraisal of nonrandomized studies—a review of recommended and commonly used tools. J. Eval. Clin. Pract. 25 (1), 44–52. 10.1111/JEP.12889 29484779

[B70] Ramaekers J. G. Kauert G. Theunissen E. L. Toennes S. W. Moeller M. R. (2009). Neurocognitive performance during acute THC intoxication in heavy and occasional cannabis users. J. Psychopharmacol. 23 (3), 266–277. 10.1177/0269881108092393 18719045

[B71] Ramaekers J. G. Kauert G. Van Ruitenbeek P. Theunissen E. L. Schneider E. Moeller M. R. (2006a). High-potency marijuana impairs executive function and inhibitory motor control. Neuropsychopharmacology 31 (10), 2296–2303. 10.1038/sj.npp.1301068 16572123

[B72] Ramaekers J. G. Mason N. L. Toennes S. W. Theunissen E. L. Amico E. (2022). Functional brain connectomes reflect acute and chronic cannabis use. Sci. Rep. 12 (1), 2449. 10.1038/s41598-022-06509-9 35165360 PMC8844352

[B73] Ramaekers J. G. Moeller M. R. van Ruitenbeek P. Theunissen E. L. Schneider E. Kauert G. (2006b). Cognition and motor control as a function of Delta9-THC concentration in serum and oral fluid: limits of impairment. Drug Alcohol Dependence 85 (2), 114–122. 10.1016/j.drugalcdep.2006.03.015 16723194

[B74] Ramaekers J. G. Theunissen E. L. De Brouwer M. Toennes S. W. Moeller M. R. Kauert G. (2011). Tolerance and cross-tolerance to neurocognitive effects of THC and alcohol in heavy cannabis users. Psychopharmacology 214 (2), 391–401. 10.1007/s00213-010-2042-1 21049267 PMC3045517

[B75] Romero-Sandoval E. A. Fincham J. E. Kolano A. L. Sharpe B. N. Alvarado-Vázquez P. A. (2018). Cannabis for chronic pain: challenges and considerations. Pharmacother. J. Hum. Pharmacol. Drug Ther. 38 (6), 651–662. 10.1002/PHAR.2115 29637590

[B76] Saccà F. Pane C. Carotenuto A. Massarelli M. Lanzillo R. Florio E. B. (2016). The use of medical-grade cannabis in patients non-responders to Nabiximols. J. Neurological Sci. 368, 349–351. 10.1016/j.jns.2016.07.059 27538663

[B77] Sainsbury B. Bloxham J. Pour M. H. Padilla M. Enciso R. (2021). Efficacy of cannabis-based medications compared to placebo for the treatment of chronic neuropathic pain: a systematic review with meta-analysis. J. Dent. Anesth. Pain Med. 21 (6), 479–506. 10.17245/jdapm.2021.21.6.479 34909469 PMC8637910

[B97] Schardt C. Adams M. B. Owens T. Keitz S. Fontelo P. (2007). Utilization of the PICO framework to improve searching PubMed for clinical questions. BMC Med. Inform. Decis. Mak. 7, 1–6. 10.1186/1472-6947-7-16 17573961 PMC1904193

[B101] Sholler D. J. Strickland J. C. Spindle T. R. Weerts E. M. Vandrey R. (2021). Sex differences in the acute effects of oral and vaporized cannabis among healthy adults. Addict. Biol. 26 (4), e12968. 10.1111/adb.12968 32985064 PMC8855307

[B78] Skumlien M. Freeman T. P. Hall D. Mokrysz C. Wall M. B. Ofori S. (2023). The effects of acute cannabis with and without cannabidiol on neural reward anticipation in adults and adolescents. Biol. Psychiatry Cognitive Neurosci. Neuroimaging 8 (2), 219–229. 10.1016/j.bpsc.2022.10.004 36642667

[B79] Solowij N. Broyd S. Greenwood L. marie van Hell H. Martelozzo D. Rueb K. (2019). A randomised controlled trial of vaporised Δ 9 -tetrahydrocannabinol and cannabidiol alone and in combination in frequent and infrequent cannabis users: acute intoxication effects. Eur. Archives Psychiatry Clin. Neurosci. 269 (1), 17–35. 10.1007/s00406-019-00978-2 30661105

[B80] Spronk D. B. De Bruijn E. R. A. van Wel J. H. P. Ramaekers J. G. Verkes R. J. (2016a). Acute effects of cocaine and cannabis on response inhibition in humans: an ERP investigation. Addict. Biol. 21 (6), 1186–1198. 10.1111/adb.12274 26037156

[B81] Spronk D. B. Van Der Schaaf M. E. Cools R. De Bruijn E. R. A. Franke B. Van Wel J. H. P. (2016b). Acute effects of cocaine and cannabis on reversal learning as a function of COMT and DRD2 genotype. Psychopharmacology 233 (2), 199–211. 10.1007/s00213-015-4141-5 26572896 PMC4700084

[B82] Spronk D. B. Verkes R. J. Cools R. Franke B. Van Wel J. H. P. Ramaekers J. G. (2016c). Opposite effects of cannabis and cocaine on performance monitoring. Eur. Neuropsychopharmacol. 26 (7), 1127–1139. 10.1016/j.euroneuro.2016.03.015 27106715

[B83] Szejko N. Fremer C. Baacke F. Ptok M. Müller-Vahl K. R. (2021). Cannabis improves stuttering: case report and interview with the patient. Cannabis Cannabinoid Res. 6 (5), 372–380. 10.1089/can.2021.0060 34314602 PMC8612409

[B84] Szejko N. Jakubovski E. Fremer C. Müller-Vahl K. R. (2019). Vaporized cannabis is effective and well-tolerated in an adolescent with tourette syndrome. Med. Cannabis Cannabinoids 2 (1), 60–63. 10.1159/000496355 34676335 PMC8489327

[B103] Tank A. Tietz T. Daldrup T. Schwender H. Hellen F. Ritz-Timme S. (2019). On the impact of cannabis consumption on traffic safety: a driving simulator study with habitual cannabis consumers. Int. J. Legal Med. 133 (5), 1411–1420. 10.1007/s00414-019-02006-3 30701315

[B100] Theunissen E. L. Heckman P. de Sousa Fernandes Perna E. B. Kuypers K. P. C. Sambeth A. Blokland A. (2015). Rivastigmine but not vardenafil reverses cannabis-induced impairment of verbal memory in healthy humans. Psychopharmacology 232, 343–353. 10.1007/s00213-014-3667-2 24998257

[B85] UNITED NATIONS OFFICE ON DRUGS AND LABOR (2021). World drug report 2020 (set of 6 booklets). Vienna: UN, Office on Drugs and Crime.

[B86] van Dam C. J. van der Schrier R. van Velzen M. van Lemmen M. Simons P. Kuijpers K. W. K. (2023). Inhaled Δ9-tetrahydrocannabinol does not enhance oxycodone-induced respiratory depression: randomised controlled trial in healthy volunteers. Br. J. Anaesth. 130 (4), 485–493. 10.1016/j.bja.2022.12.018 36725378

[B87] Van De Donk T. Niesters M. Kowal M. A. Olofsen E. Dahan A. Van Velzen M. (2019). An experimental randomized study on the analgesic effects of pharmaceutical-grade cannabis in chronic pain patients with fibromyalgia. Pain 160 (4), 860–869. 10.1097/j.pain.0000000000001464 30585986 PMC6430597

[B98] Vermetten E. de Wit J. (2023). Medical cannabis for chronic posttraumatic stress disorder in dutch veterans: a health care evaluation. Med. Res. Arch. 11 (11). 10.18103/mra.v11i11.4503

[B88] Vulfsons S. Ognitz M. Bar-Sela G. Raz-Pasteur A. Eisenberg E. (2020). Cannabis treatment in hospitalized patients using the SYQE inhaler: results of a pilot open-label study. Palliat. Support. Care 18 (1), 12–17. 10.1017/S147895151900021X 31196236

[B89] Wall M. B. Freeman T. P. Hindocha C. Demetriou L. Ertl N. Freeman A. M. (2022). Individual and combined effects of cannabidiol and Δ9-tetrahydrocannabinol on striato-cortical connectivity in the human brain. J. Psychopharmacol. 36 (6), 732–744. 10.1177/02698811221092506 35596578 PMC9150138

[B90] Wall M. B. Pope R. Freeman T. P. Kowalczyk O. S. Demetriou L. Mokrysz C. (2019). Dissociable effects of cannabis with and without cannabidiol on the human brain’s resting-state functional connectivity. J. Psychopharmacol. 33 (7), 822–830. 10.1177/0269881119841568 31013455

[B92] Xiao K. B. Grennell E. Ngoy A. George T. P. Le Foll B. Hendershot C. S. (2023). Cannabis self-administration in the human laboratory: a scoping review of *ad libitum* studies. Psychopharmacology 240 (7), 1393–1415. 10.1007/s00213-023-06360-4 37157001 PMC10272254

[B93] Zafar R. Schlag A. Nutt D. (2020). Ending the pain of children with severe epilepsy? An audit of the impact of medical cannabis in 10 patients. Drug Sci. Policy Law 6, 205032452097448. 10.1177/2050324520974487

[B94] Zafar R. Schlag A. Phillips L. Nutt D. J. (2021). Medical cannabis for severe treatment resistant epilepsy in children: a case-series of 10 patients. BMJ Paediatr. Open 5 (1), e001234. 10.1136/bmjpo-2021-001234

[B95] Zamarripa C. A. Vandrey R. Spindle T. R. (2022). Factors that impact the pharmacokinetic and pharmacodynamic effects of cannabis: a review of human laboratory studies. Curr. Addict. Rep. 9 (4), 608–621. 10.1007/s40429-022-00429-4

[B96] Zhornitsky S. Pelletier J. Assaf R. Giroux S. Li C. shan R. Potvin S. (2021). Acute effects of partial CB1 receptor agonists on cognition – a meta-analysis of human studies. Prog. Neuro-Psychopharmacology Biol. Psychiatry 104, 110063. 10.1016/j.pnpbp.2020.110063 32791166

