# Supplementary material for: The effects of standardized cannabis products in healthy volunteers and patients: a systematic literature review
Source: Front Pharmacol. 2024 Oct 17;15:1411631. doi: 10.3389/fphar.2024.1411631 (PMC11524849; doi:10.3389/fphar.2024.1411631)
Supplement: Supplementary file 1 [file DataSheet1.docx]

**Supplemental Materials**

**Overview of studies included in this systematic review (N=60)**

Studies found through the PubMed search (n=49)

Almog, S., Aharon-Peretz, J., Vulfsons, S., Ogintz, M., Abalia, H., Lupo, T., Hayon, Y., & Eisenberg, E. (2020). The pharmacokinetics, efficacy, and safety of a novel selective-dose cannabis inhaler in patients with chronic pain: A randomized, double-blinded, placebo-controlled trial. *European Journal of Pain (United Kingdom)*, *24*(8), 1505–1516. <https://doi.org/10.1002/ejp.1605>

Arkell, T. R., Vinckenbosch, F., Kevin, R. C., Theunissen, E. L., McGregor, I. S., & Ramaekers, J. G. (2020). Effect of Cannabidiol and ?9-Tetrahydrocannabinol on Driving Performance: A Randomized Clinical Trial. *JAMA - Journal of the American Medical Association*, *324*(21), 2177–2186. <https://doi.org/10.1001/jama.2020.21218>

Baraldi, C., Lo Castro, F., Negro, A., Ferrari, A., Cainazzo, M. M., Pani, L., & Guerzoni, S. (2022). Oral Cannabinoid Preparations for the Treatment of Chronic Migraine: A Retrospective Study. *Pain Medicine (United States)*, *23*(2), 396–402. <https://doi.org/10.1093/pm/pnab245>

Battistella, G., Fornari, E., Thomas, A., Mall, J. F., Chtioui, H., Appenzeller, M., Annoni, J. M., Favrat, B., Maeder, P., & Giroud, C. (2013). Weed or Wheel! fMRI, Behavioural, and Toxicological Investigations of How Cannabis Smoking Affects Skills Necessary for Driving. *PLoS ONE*, *8*(1). <https://doi.org/10.1371/journal.pone.0052545>

Dar, S. (2021). Treating pain related to Ehlers-Danlos syndrome with medical cannabis. *BMJ Case Reports*, *14*(7). <https://doi.org/10.1136/bcr-2021-242568>

de Wit, J., & Vermetten, E. (2023). Medicinal Cannabis for Chronic Posttraumatic Stress Disorder in Dutch Veterans: A Health Care Evaluation. *Medical Research Archives,* 11(11). *OPEN ACCESS*. (2023). <https://doi.org/10.18103/m>

Eisenberg, E., Ogintz, M., & Almog, S. (2014). The pharmacokinetics, efficacy, safety, and ease of use of a novel portable metered-dose cannabis inhaler in patients with chronic neuropathic pain: A phase 1a study. *Journal of Pain and Palliative Care Pharmacotherapy*, *28*(3), 216–225. <https://doi.org/10.3109/15360288.2014.941130>

Engels, F. K., de Jong, F. A., Sparreboom, A., Mathot, R. A. A., Loos, W. J., Kitzen, J. J. E. M., de Bruijn, P., Verweij, J., & Mathijssen, R. H. J. (2007). Medicinal Cannabis Does Not Influence the Clinical Pharmacokinetics of Irinotecan and Docetaxel. *The Oncologist*, *12*(3), 291–300. <https://doi.org/10.1634/theoncologist.12-3-291>

Englund, A., Oliver, D., Chesney, E., Chester, L., Wilson, J., Sovi, S., De Micheli, A., Hodsoll, J., Fusar-Poli, P., Strang, J., Murray, R. M., Freeman, T. P., & McGuire, P. (2023). Does cannabidiol make cannabis safer? A randomised, double-blind, cross-over trial of cannabis with four different CBD:THC ratios. *Neuropsychopharmacology*, *48*(6), 869–876. <https://doi.org/10.1038/s41386-022-01478-z>

Fabritius, M., Chtioui, H., Battistella, G., Annoni, J. M., Dao, K., Favrat, B., Fornari, E., Lauer, E., Maeder, P., & Giroud, C. (2013). Comparison of cannabinoid concentrations in oral fluid and whole blood between occasional and regular cannabis smokers prior to and after smoking a cannabis joint. *Analytical and Bioanalytical Chemistry*, *405*(30), 9791–9803. <https://doi.org/10.1007/s00216-013-7412-1>

Freeman, A. M., Mokrysz, C., Hindocha, C., Lawn, W., Morgan, C. J. A., Freeman, T. P., Saunders, R., & Curran, H. V. (2021). Does variation in trait schizotypy and frequency of cannabis use influence the acute subjective, cognitive and psychotomimetic effects of delta-9-tetrahydrocannabinol? A mega-analysis. *Journal of Psychopharmacology*, *35*(7), 804–813. <https://doi.org/10.1177/0269881120959601>

Giorgi, V., Bongiovanni, S., Atzeni, F., Marotto, D., Salaffi, F., & Sarzi-Puttini, P. (2020). Adding medical cannabis to standard analgesic treatment for fibromyalgia: a prospective observational study. *Clinical and Experimental Rheumatology*, 38 Suppl 123(1):53-59. Epub 2020 Feb 5. PMID: 32116208.

Hunault, C. C., Böcker, K. B. E., Stellato, R. K., Kenemans, J. L., De Vries, I., & Meulenbelt, J. (2014). Acute subjective effects after smoking joints containing up to 69 mg Δ9-tetrahydrocannabinol in recreational users: A randomized, crossover clinical trial. *Psychopharmacology*, *231*(24), 4723–4733. <https://doi.org/10.1007/s00213-014-3630-2>

Hunault, C. C., Mensinga, T. T., Böcker, K. B. E., Schipper, C. M. A., Kruidenier, M., Leenders, M. E. C., De Vries, I., & Meulenbelt, J. (2009). Cognitive and psychomotor effects in males after smoking a combination of tobacco and cannabis containing up to 69 mg delta-9-tetrahydrocannabinol (THC). *Psychopharmacology*, *204*(1), 85–94. <https://doi.org/10.1007/s00213-008-1440-0>

Hunault, C. C., Mensinga, T. T., De Vries, I., Kelholt-Dijkman, H. H., Hoek, J., Kruidenier, M., Leenders, M. E. C., & Meulenbelt, J. (2008). Delta-9-tetrahydrocannabinol (THC) serum concentrations and pharmacological effects in males after smoking a combination of tobacco and cannabis containing up to 69 mg THC. *Psychopharmacology*, *201*(2), 171–181. <https://doi.org/10.1007/s00213-008-1260-2>

Hutten, N. R. P. W., Arkell, T. R., Vinckenbosch, F., Schepers, J., Kevin, R. C., Theunissen, E. L., Kuypers, K. P. C., McGregor, I. S., & Ramaekers, J. G. (2022). Cannabis containing equivalent concentrations of delta-9-tetrahydrocannabinol (THC) and cannabidiol (CBD) induces less state anxiety than THC-dominant cannabis. *Psychopharmacology*, *239*(11), 3731–3741. <https://doi.org/10.1007/s00213-022-06248-9>

Jakubovski, E., & Müller-Vahl, K. (2017). Speechlessness in Gilles de la Tourette Syndrome: Cannabis-based medicines improve severe vocal blocking tics in two patients. *International Journal of Molecular Sciences*, *18*(8). <https://doi.org/10.3390/ijms18081739>

Kloft, L., Otgaar, H., Blokland, A., Monds, L. A., Toennes, S. W., Loftus, E. F., & Ramaekers, J. G. (2020). *Cannabis increases susceptibility to false memory*. *117*(9), 4585–4589. <https://doi.org/10.1073/pnas.1920162117/-/DCSupplemental>

Kowal, M. A., Hazekamp, A., Colzato, L. S., Van Steenbergen, H., Van Der Wee, N. J. A., Durieux, J., Manai, M., & Hommel, B. (2015). Cannabis and creativity: Highly potent cannabis impairs divergent thinking in regular cannabis users. *Psychopharmacology*, *232*(6), 1123–1134. <https://doi.org/10.1007/s00213-014-3749-1>

Kowal, M. A., van Steenbergen, H., Colzato, L. S., Hazekamp, A., van der Wee, N. J. A., Manai, M., Durieux, J., & Hommel, B. (2015). Dose-dependent effects of cannabis on the neural correlates of error monitoring in frequent cannabis users. *European Neuropsychopharmacology*, *25*(11), 1943–1953. <https://doi.org/10.1016/j.euroneuro.2015.08.001>

Lawn, W., Freeman, T. P., Pope, R. A., Joye, A., Harvey, L., Hindocha, C., Mokrysz, C., Moss, A., Wall, M. B., Bloomfield, M. A., Das, R. K., Morgan, C. J., Nutt, D. J., & Curran, H. V. (2016). Acute and chronic effects of cannabinoids on effort-related decision-making and reward learning: an evaluation of the cannabis ‘amotivational’ hypotheses. *Psychopharmacology*, *233*(19–20), 3537–3552. <https://doi.org/10.1007/s00213-016-4383-x>

Lawn, W., Trinci, K., Mokrysz, C., Borissova, A., Ofori, S., Petrilli, K., Bloomfield, M., Haniff, Z. R., Hall, D., Fernandez-Vinson, N., Wang, S., Englund, A., Chesney, E., Wall, M. B., Freeman, T. P., & Curran, H. V. (2023). The acute effects of cannabis with and without cannabidiol in adults and adolescents: A randomised, double-blind, placebo-controlled, crossover experiment. *Addiction*, *118*(7), 1282–1294. <https://doi.org/10.1111/add.16154>

Mason, N. L., Theunissen, E. L., Hutten, N. R. P. W., Tse, D. H. Y., Toennes, S. W., Jansen, J. F. A., Stiers, P., & Ramaekers, J. G. (2021). Reduced responsiveness of the reward system is associated with tolerance to cannabis impairment in chronic users. *Addiction Biology*, *26*(1). <https://doi.org/10.1111/adb.12870>

Mason, N. L., Theunissen, E. L., Hutten, N. R. P. W., Tse, D. H. Y., Toennes, S. W., Stiers, P., & Ramaekers, J. G. (2019). Cannabis induced increase in striatal glutamate associated with loss of functional corticostriatal connectivity. *European Neuropsychopharmacology*, *29*(2), 247–256. <https://doi.org/10.1016/j.euroneuro.2018.12.003>

Mokrysz, C., Freeman, T. P., Korkki, S., Griffiths, K., & Curran, H. V. (2016). Are adolescents more vulnerable to the harmful effects of cannabis than adults? A placebo-controlled study in human males. *Translational Psychiatry*, *6*(11). <https://doi.org/10.1038/tp.2016.225>

Mokrysz, C., Shaban, N. D. C., Freeman, T. P., Lawn, W., Pope, R. A., Hindocha, C., Freeman, A., Wall, M. B., Bloomfield, M. A. P., Morgan, C. J. A., Nutt, D. J., & Curran, H. V. (2021). Acute effects of cannabis on speech illusions and psychotic-like symptoms: two studies testing the moderating effects of cannabidiol and adolescence. *Psychological Medicine*, *51*(12), 2134–2142. <https://doi.org/10.1017/S0033291720001038>

Nunnari, P., Ladiana, N., Ceccarelli, G., & Notato, P. (2022). Long-term Cannabis-based oil therapy and pain medications prescribing patterns: an Italian observational study. *European Review for Medical and Pharmacological Sciences*, 26: 1224-1234.

Palmieri, B., Laurino, C., & Vadalà, M. (2019). Spontaneous, anecdotal, retrospective, open-label study on the efficacy, safety and tolerability of cannabis galenical preparation (Bedrocan). *International Journal of Pharmacy Practice*, *27*(3), 264–270. <https://doi.org/10.1111/ijpp.12514>

Palmieri, B., & Vadalà, M. (2023). Oral thc: cbd cannabis extract in main symptoms of Alzheimer disease: agitation and weight loss. *Clinica Terapeutica*, *174*(1), 53–60. <https://doi.org/10.7417/CT.2023.2497>

Pane, C., & Saccà, F. (2020). The use of medical grade cannabis in Italy for drug-resistant epilepsy: a case series. *Neurological Sciences*, *41*(3), 695–698. <https://doi.org/10.1007/s10072-019-04162-1>

Poli, P., Crestani, F., Salvadori, C., Valenti, I., & Sannino, C. (2018). Medical cannabis in patients with chronic pain: Effect on pain relief, pain disability, and psychological aspects. A prospective non randomized single arm clinical trial. *Clinica Terapeutica*, *169*(3), E102–E107. <https://doi.org/10.7417/T.2018.2062>

Ramaekers, J. G., Kauert, G., Theunissen, E. L., Toennes, S. W., & Moeller, M. R. (2009). Neurocognitive performance during acute THC intoxication in heavy and occasional cannabis users. *Journal of Psychopharmacology*, *23*(3), 266–277. <https://doi.org/10.1177/0269881108092393>

Ramaekers, J. G., Kauert, G., Van Ruitenbeek, P., Theunissen, E. L., Schneider, E., & Moeller, M. R. (2006). High-potency marijuana impairs executive function and inhibitory motor control. *Neuropsychopharmacology*, *31*(10), 2296–2303. <https://doi.org/10.1038/sj.npp.1301068>

Ramaekers, J. G., Mason, N. L., Toennes, S. W., Theunissen, E. L., & Amico, E. (2022). Functional brain connectomes reflect acute and chronic cannabis use. *Scientific Reports*, *12*(1). <https://doi.org/10.1038/s41598-022-06509-9>

Ramaekers, J. G., Moeller, M. R., van Ruitenbeek, P., Theunissen, E. L., Schneider, E., & Kauert, G. (2006). Cognition and motor control as a function of Δ9-THC concentration in serum and oral fluid: Limits of impairment. *Drug and Alcohol Dependence*, *85*(2), 114–122. <https://doi.org/10.1016/j.drugalcdep.2006.03.015>

Ramaekers, J. G., Theunissen, E. L., De Brouwer, M., Toennes, S. W., Moeller, M. R., & Kauert, G. (2011). Tolerance and cross-tolerance to neurocognitive effects of THC and alcohol in heavy cannabis users. *Psychopharmacology*, *214*(2), 391–401. <https://doi.org/10.1007/s00213-010-2042-1>

Saccà, F., Pane, C., Carotenuto, A., Massarelli, M., Lanzillo, R., Florio, E. B., & Brescia Morra, V. (2016). The use of medical-grade cannabis in patients non-responders to Nabiximols. In *Journal of the Neurological Sciences* (Vol. 368, pp. 349–351). Elsevier B.V. <https://doi.org/10.1016/j.jns.2016.07.059>

Skumlien, M., Freeman, T. P., Hall, D., Mokrysz, C., Wall, M. B., Ofori, S., Petrilli, K., Trinci, K., Borissova, A., Fernandez-Vinson, N., Langley, C., Sahakian, B. J., Curran, H. V., & Lawn, W. (2023). The Effects of Acute Cannabis With and Without Cannabidiol on Neural Reward Anticipation in Adults and Adolescents. *Biological Psychiatry: Cognitive Neuroscience and Neuroimaging*, *8*(2), 219–229. <https://doi.org/10.1016/j.bpsc.2022.10.004>

Spronk, D. B., De Bruijn, E. R. A., van Wel, J. H. P., Ramaekers, J. G., & Verkes, R. J. (2016). Acute effects of cocaine and cannabis on response inhibition in humans: an ERP investigation. *Addiction Biology*, *21*(6), 1186–1198. <https://doi.org/10.1111/adb.12274>

Spronk, D. B., Van Der Schaaf, M. E., Cools, R., De Bruijn, E. R. A., Franke, B., Van Wel, J. H. P., Ramaekers, J. G., & Verkes, R. J. (2016). Acute effects of cocaine and cannabis on reversal learning as a function of COMT and DRD2 genotype. *Psychopharmacology*, *233*(2), 199–211. <https://doi.org/10.1007/s00213-015-4141-5>

Spronk, D. B., Verkes, R. J., Cools, R., Franke, B., Van Wel, J. H. P., Ramaekers, J. G., & De Bruijn, E. R. A. (2016). Opposite effects of cannabis and cocaine on performance monitoring. *European Neuropsychopharmacology*, *26*(7), 1127–1139. <https://doi.org/10.1016/j.euroneuro.2016.03.015>

Szejko, N., Fremer, C., Baacke, F., Ptok, M., & Müller-Vahl, K. R. (2021). Cannabis Improves Stuttering: Case Report and Interview with the Patient. In *Cannabis and Cannabinoid Research* (Vol. 6, Issue 5, pp. 372–380). Mary Ann Liebert Inc. <https://doi.org/10.1089/can.2021.0060>

Tank, A., Tietz, T., Daldrup, T., Schwender, H., Hellen, F., Ritz-Timme, S., & Hartung, B. (2019). On the impact of cannabis consumption on traffic safety: a driving simulator study with habitual cannabis consumers. *International Journal of Legal Medicine*, *133*(5), 1411–1420. <https://doi.org/10.1007/s00414-019-02006-3>

Theunissen, E. L., Heckman, P., De Sousa Fernandes Perna, E. B., Kuypers, K. P. C., Sambeth, A., Blokland, A., Prickaerts, J., Toennes, S. W., & Ramaekers, J. G. (2015). Rivastigmine but not vardenafil reverses cannabis-induced impairment of verbal memory in healthy humans. *Psychopharmacology*, *232*(2), 343–353. <https://doi.org/10.1007/s00213-014-3667-2>

van Dam, C. J., van der Schrier, R., van Velzen, M., van Lemmen, M., Simons, P., Kuijpers, K. W. K., Jansen, S., Kowal, M. A., Olofsen, E., Kramers, C., Dahan, A., & Niesters, M. (2023). Inhaled Δ9-tetrahydrocannabinol does not enhance oxycodone-induced respiratory depression: randomised controlled trial in healthy volunteers. *British Journal of Anaesthesia*, *130*(4), 485–493. <https://doi.org/10.1016/j.bja.2022.12.018>

Van De Donk, T., Niesters, M., Kowal, M. A., Olofsen, E., Dahan, A., & Van Velzen, M. (2019). An experimental randomized study on the analgesic effects of pharmaceutical-grade cannabis in chronic pain patients with fibromyalgia. *Pain*, *160*(4), 860–869. <https://doi.org/10.1097/j.pain.0000000000001464>

Vulfsons, S., Ognitz, M., Bar-Sela, G., Raz-Pasteur, A., & Eisenberg, E. (2020). Cannabis treatment in hospitalized patients using the SYQE inhaler: Results of a pilot open-label study. *Palliative and Supportive Care*, *18*(1), 12–17. <https://doi.org/10.1017/S147895151900021X>

Wall, M. B., Freeman, T. P., Hindocha, C., Demetriou, L., Ertl, N., Freeman, A. M., Jones, A. P. M., Lawn, W., Pope, R., Mokrysz, C., Solomons, D., Statton, B., Walker, H. R., Yamamori, Y., Yang, Z., Yim, J. L. L., Nutt, D. J., Howes, O. D., Curran, H. V., & Bloomfield, M. A. P. (2022). Individual and combined effects of cannabidiol and Δ9-tetrahydrocannabinol on striato-cortical connectivity in the human brain. *Journal of Psychopharmacology*, *36*(6), 732–744. <https://doi.org/10.1177/02698811221092506>

Wall, M. B., Pope, R., Freeman, T. P., Kowalczyk, O. S., Demetriou, L., Mokrysz, C., Hindocha, C., Lawn, W., Bloomfield, M. A. P., Freeman, A. M., Feilding, A., Nutt, D., & Curran, H. V. (2019). Dissociable effects of cannabis with and without cannabidiol on the human brain’s resting-state functional connectivity. *Journal of Psychopharmacology*, *33*(7), 822–830. <https://doi.org/10.1177/0269881119841568>

Studies found through examination of references in other papers (n=11)

Arkell, T. R., Kevin, R. C., Vinckenbosch, F., Lintzeris, N., Theunissen, E., Ramaekers, J. G., & McGregor, I. S. (2022). Sex differences in acute cannabis effects revisited: Results from two randomized, controlled trials. *Addiction Biology*, *27*(2). <https://doi.org/10.1111/adb.13125>

Aviram, J., Atzmony, D., & Eisenberg, E. (2022). Long-term effectiveness and safety of medical cannabis administered through the metered-dose Syqe Inhaler. *Pain Reports*, *7*(3), E1011. <https://doi.org/10.1097/PR9.0000000000001011>

Brenneisen, R., Meyer, P., Chtioui, H., Saugy, M., & Kamber, M. (2010). Plasma and urine profiles of Δ9-tetrahydrocannabinol and its metabolites 11-hydroxy-Δ9-tetrahydrocannabinol and 11-nor-9-carboxy-Δ9-tetrahydrocannabinol after cannabis smoking by male volunteers to estimate recent consumption by athletes. *Analytical and Bioanalytical Chemistry*, *396*(7), 2493–2502. <https://doi.org/10.1007/s00216-009-3431-3>

Chester, L. A., Englund, A., Chesney, E., Oliver, D., Wilson, J., Sovi, S., Dickens, A. M., Oresic, M., Linderman, T., Hodsoll, J., Minichino, A., Strang, J., Murray, R. M., Freeman, T. P., & McGuire, P. (2022). Effects of Cannabidiol and Delta-9-Tetrahydrocannabinol on Plasma Endocannabinoid Levels in Healthy Volunteers: A Randomized Double-Blind Four-Arm Crossover Study. *Cannabis and Cannabinoid Research*. <https://doi.org/10.1089/can.2022.0174>

de Bruijn, S. E. M., de Graaf, C., Witkamp, R. F., & Jager, G. (2017). Explorative Placebo-Controlled Double-Blind Intervention Study with Low Doses of Inhaled Δ9-Tetrahydrocannabinol and Cannabidiol Reveals No Effect on Sweet Taste Intensity Perception and Liking in Humans. *Cannabis and Cannabinoid Research*, *2*(1), 114–122. <https://doi.org/10.1089/can.2017.0018>

Hupli, A. M. M. (2019). Medical Cannabis for Adult Attention Deficit Hyperactivity Disorder: Sociological Patient Case Report of Cannabinoid Therapeutics in Finland. *Medical Cannabis and Cannabinoids*, *1*(2), 112–118. <https://doi.org/10.1159/000495307>

Mazza, M. (2021). Medical cannabis for the treatment of fibromyalgia syndrome: a retrospective, open-label case series. *Journal of Cannabis Research*, *3*(1). <https://doi.org/10.1186/s42238-021-00060-6>

Oliver, D., Englund, A., Chesney, E., Chester, L., Wilson, J., Sovi, S., Wigroth, S., Hodsoll, J., Strang, J., Murray, R. M., Freeman, T. P., Fusar-Poli, P., & McGuire, P. (2023). Cannabidiol does not attenuate acute delta-9-tetrahydrocannabinol-induced attentional bias in healthy volunteers: A randomised, double-blind, cross-over study. *Addiction*. <https://doi.org/10.1111/add.16353>

Szejko, N., Jakubovski, E., Fremer, C., & Müller-Vahl, K. R. (2019). Vaporized Cannabis Is Effective and Well-Tolerated in an Adolescent with Tourette Syndrome. *Medical Cannabis and Cannabinoids*, *2*(1), 60–64. <https://doi.org/10.1159/000496355>

Zafar, R., Schlag, A., & Nutt, D. (2020). Ending the pain of children with severe epilepsy? An audit of the impact of medical cannabis in 10 patients. *Drug Science, Policy and Law*, *6*, 205032452097448. <https://doi.org/10.1177/2050324520974487>

Zafar, R., Schlag, A., Phillips, L., & Nutt, D. J. (2021). Medical cannabis for severe treatment resistant epilepsy in children: A case-series of 10 patients. *BMJ Paediatrics Open*, *5*(1). <https://doi.org/10.1136/bmjpo-2021-001234>


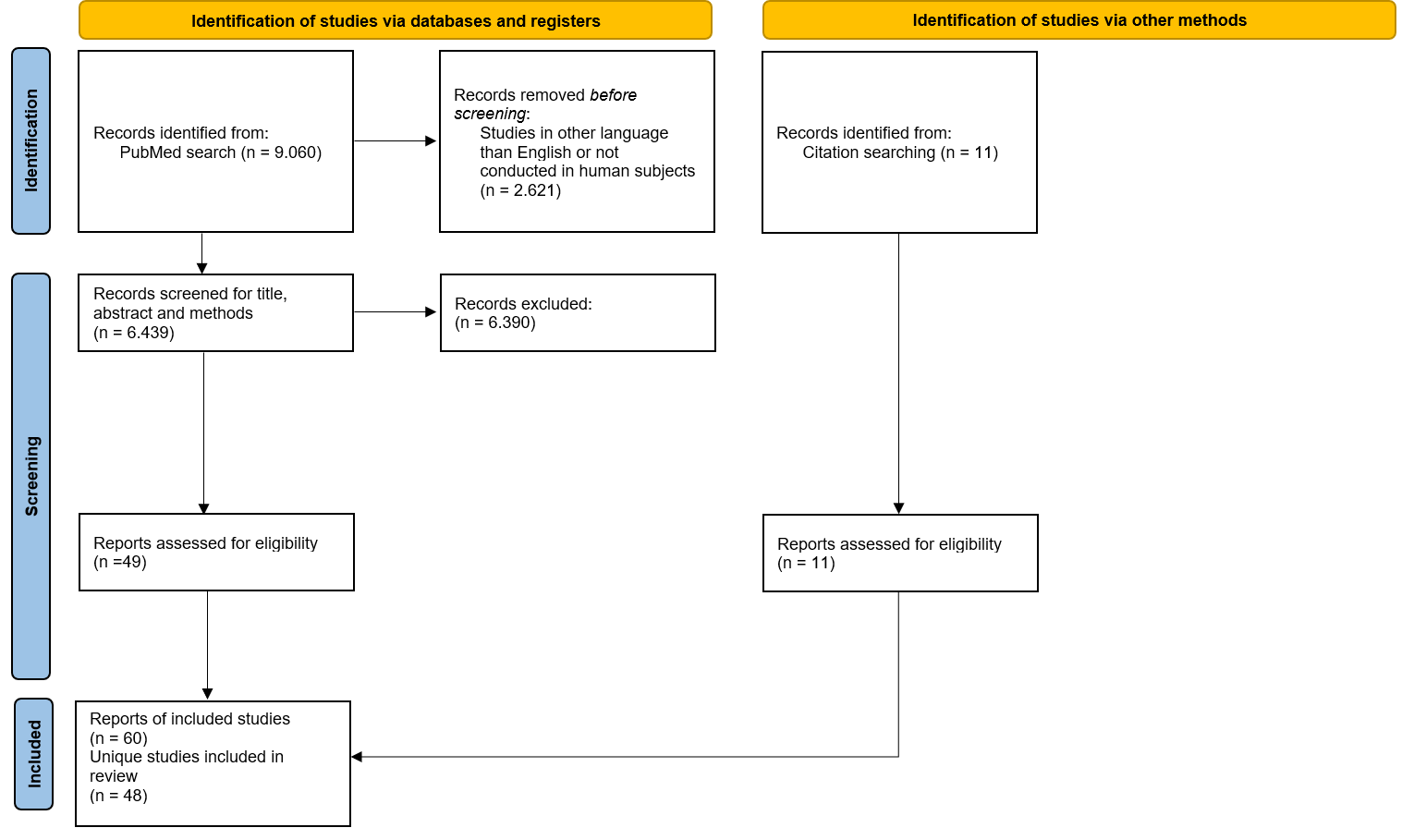


**Figure S1.** PRISMA flow diagram for systematic review

**Table S1.** Overview of side effects and drop-out rates

| First Author | Start  sample | Drop-out | Analyzing  sample | Bedrocan | Bediol | Bedrolite | Combination treatment | Placebo |
| --- | --- | --- | --- | --- | --- | --- | --- | --- |
| van de Donk et al. (2019) | 25 | 1 = unknown reasons 3 = nausea during visit** 1 = fear of needles | 20 | 16 = drug high  14 = coughing  5 = bad taste  3 = nausea  3 = dizzy  2 = sore throat  1 = headache  1= sleepy | 16 = drug high  14 = coughing  7 = sore throat  6 = bad taste  6 = nausea  4 = dizzy  2 = headache  1 = dyspnoea | 13 = coughing 8 = drug high 5 = bad taste  3 = headache  2 = dizzy  1 = sore throat  1 = nausea  1 = vomiting  1 = sleepy | NA | 2 = drug high 1 = headache |
| Almog et al. (2020) | 27 | 1 = did not complete all sessions due to technical failure 1 = consent withdrawal | 27 | **0.5 mg** 12 = drug high  8 = dry mouth 7 = pain 7 = weakness  7 = restlessness  6 = sleepiness 4 = cough 2 = dizziness 2 = nausea  2 = BP reduced **1.0 mg** 16 = drug high  11 = cough  8 = dizziness 6 = weakness  5 = dry mouth 4 = sleepiness 4 = nausea  3 = pain 3 = restlessness  2 = BP reduced 2 = hunger | NA | NA | NA | 9 = drug high  7 = restlessness 6 = pain 5 = weakness  3 = hunger 2 = dry mouth 2 = sleepiness 2 = cough 2 = nausea  2 = BP reduced 1 = dizziness |
| Aviram et al. (2022) | 215 | **excluded from analysis** 25 = stopped treatment before achieving a balanced regimen 25 = pilot program 13 = stopped during titration period 9 = requested to not respond to questions **reasons for stopping treatment** 8 = worsening of medical condition 7 = financial reasons 5 = ineffectiveness of treatment 3 = deceased (unrelated to treatment) 3 = no longer need treatment 1 = adverse event** 1 = regulatory reasons | 143 | 25 = dizziness 15 = headache 11 = sleepiness 8 = anxiety 6 = dry mouth 4 = nausea 4 = cough 3 = heartburn 3 = confusion 3 = concentration impairment 2 = memory impairment 2 = restlessness 2 = palpitations 1 = vomiting 1 = tinnitus 1 = muscle pain | NA | NA | NA | NA |
| Baraldi et al. (2021)* | 32 | after 6 months  2 = vertigo** 2 = NR | 32 | NA | NA | NA | 10 = drowsiness 2 = postural instability 1 = vertigo 1 = weight gain **baseline (n=32)** 30 = nausea and/or vomiting 19 = photophobia and/or phonophobia **3 months (n=32)** 20 = nausea and/or vomiting 19 = photophobia and/or phonophobia **6 months (n=28)** 19 = nausea and/or vomiting 19 = photophobia and/or phonophobia | NA |
| Eisenberg et al. (2014) | 10 | 2 = failed device | 8 | 7 = lightheadedness for the first 10 minutes | NA | NA | NA | NA |
| Giorgi et al. (2020) | 102 | 25 = lost to follow-up 3 = lack of benefit effect 6 = adverse events (mainly nausea, palpitations and dizziness)** 2 = high cost of treatment | 66 | NA | NA | NA | 14 = dizziness  11= sleepiness  8 = palpitations  6 = nausea 6 = xerostomia  5 = blurred vision 5 = stypsis 3 = headache 3 = hunger 1 = dyspnea | NA |
| Mazza et al. (2021)* | 38 | **before start of the treatment** 1 = did not start due to unpleasant scent 1 = difficulty to retrieve cannabis 1 = long period of hospitalization following prescription **1 month follow-up (n=30)** 4 = vomiting after intake** 1 = experienced several side effects** **3 months follow-up (n=18)** 12 = different side effects** **12 months follow-up (n=12)** 6 = personal reasons, moving or pharmacy no longer supplied cannabis | 35 | NA | NA | NA | **after one day to 1 month (n=35)** 13 = mental confusion 5 = nausea/vomiting 5 = vertigo/dizziness 5 = restlessness/irritation 4 = palpitations 4 = somnolence 2 = dry mouth 2 = insomnia 1 = nightmares 1 = increased hunger 1 = euphoria 1 = headache 1 = increased sexual drive 1 = abdominal distension 1 = erect nipples 1 = fatigue 1 = anger **after 3 months (n=18)** 1 = headache 1 = decreased creativity **after 12 months (n=12)** 2 = memory impairment | NA |
| Nunnari et al. (2022)* | 186 | 6 = different formulations 20 = discontinuation of cannabis-based oil before six months  104 = lack of a follow-up visit after at least six months from the first consumption | 56 | NA | NA | NA | NR | NA |
| Palmieri et al. (2019) | 20 | NA | 20 | 3 = somnolence | NA | NA | NA | NA |
| Poli et al. (2018) | 338 | **1 month follow-up** 79 = inefficacy 33 = side effects (sleepiness 30%; mental confusion 25%)** | 338 | 10 = sleepiness 8 = mental confusion 15 = other side effects | NA | NA | NA | NA |
| Vulfsons et al. (2020) | 22 | 1 = preferred not to use the inhaler | 21 | 3 = mild cough after  inhalation | NA | NA | NA | NA |
| Sacca et al. (2016) | 13 | 1 = dizziness**  1 = drug cost | 13 | 3 = dizziness | NA | NA | NA | NA |
| Engels et al. (2007) | 24 | NA | 24 | NR | NA | NA | NA | NA |
| Zafar et al. (2020) | 10 | NA | 10 | NA | NA | NA | No adverse side effects occurred | NA |
| Zafar et al. (2021)* | 10 | NA | 10 | NA | NA | NA | No adverse side effects occurred | NA |
| Pane et al. (2020) | 5 | NA | 5 | NA | NA | NA | 1 = panic attack | NA |
| Palmieri et al. (2023) | 30 | NA | 30 | No adverse side effects occurred | NA | NA | NA | NA |
| De Wit et al. (2023) | 18 | 3 = discontinued cannabis use | 18 | NA | NA | NA | 4 = drug high 1 = starving feeling anxious, depressed, or drowsy at times | NA |

*Combination treatment with medicinal cannabis from different companies, only Bedrocan products and dosing are reported. NR = Not Reported; NA = Not apllicable DB = Double Blind; PC = Placebo Controlled; Ra = Randomized; Cr = Crossover; OL = Open-Label; RE = Retrospective; PR = Prospective; QU = Qualitative. **drop-out related to side effects
